# First contact with greater gravity: Moss plants adapted via enhanced photosynthesis mediated by AP2/ERF transcription factors

**DOI:** 10.1126/sciadv.ado8664

**Published:** 2025-07-16

**Authors:** Yuko T. Hanba, Thi Huong Do, Kaori Takemura, Sakihito Kitajima, Alisa Vyacheslavova, Miyu Takata, Marcel Pascal Beier, Maki Yokoi, Akihisa Shinozawa, Ayuko Maeda, Yutaro Yasui, Naoya Sakaguchi, Ryuji Kameishi, Rina Watanabe, Souma Okugawa, Ryota Ozaki, Seika Hirai, Hiroyuki Kamachi, Atsushi Kume, Ichirou Karahara, Yoichi Sakata, Yusuke Onoda, Tomomichi Fujita

**Affiliations:** ^1^ Faculty of Applied Biology, Kyoto Institute of Technology, Matsugasaki, Sakyo-ku, Kyoto 606-8585, Japan.; ^2^ Graduate School of Life Science, Hokkaido University, Kita 10 Nishi 8, Kita-ku, Sapporo 060-0810, Japan.; ^3^ Department of Applied Biology, Kyoto Institute of Technology, Matsugasaki, Sakyo-ku, Kyoto 606-8585, Japan.; ^4^ Department of Biology, Hokkaido University, Kita 10 Nishi 8, Kita-ku, Sapporo 060-0810, Japan.; ^5^ Institute for the Advancement of Higher Education, Hokkaido University, Kita-ku, Sapporo 060-0810, Japan.; ^6^ Faculty of Science, Hokkaido University, Kita-ku, Sapporo 060-0810, Japan.; ^7^ Department of BioScience, Tokyo University of Agriculture, 1-1-1, Sakuragaoka, Setagaya-ku, Tokyo 156-8502, Japan.; ^8^ Department of Biobased Materials Science, Kyoto Institute of Technology, Matsugasaki, Sakyo-ku, Kyoto 606-8585, Japan.; ^9^ Faculty of Science, University of Toyama, 3190 Gofuku, Toyama 930-8555, Japan.; ^10^ Faculty of Agriculture, Kyushu University, 744 Motooka, Nishi-ku, Fukuoka 819-0395, Japan.; ^11^ Graduate School of Agriculture, Kyoto University, Kitashirakawa-Oiwake-cho, Sakyo-ku, Kyoto 606-8502, Japan.

## Abstract

The emergence of land plants required adaptations to altered water availability and increased effective gravity. Bryophytes underwent major changes in physiology, anatomy, and growth during their emergence onto land. However, the link between gravity, photosynthesis, and genetic control remains unclear. Here, we show that leaf carbon dioxide diffusion enhanced photosynthesis in the model moss *Physcomitrium patens* under increased gravity (6 and 10 times Earth’s gravity), driven by increases in plant (gametophore) number and chloroplast size. RNA sequencing analysis showed that 10 gravity up-regulated several species-specific APETALA2/ethylene-responsive factor (AP2/ERF) transcription factors. Overexpression of one such *AP2/ERF*, *ISSUNBOSHI1* (*IBSH1*; gene ID = *Pp3c1_32440*), in *P. patens* phenocopied plants grown at 10 gravity, and the dominant repressor form of IBSH1 suppressed 10 gravity responses. These results provide evidence that the proliferation of AP2/ERF transcription factors and the establishment of a notable gene network may have been important in adaptation to the terrestrial environment during land plant evolution.

## Teaser

Mosses adapt to greater gravity via enhanced photosynthesis mediated by AP2/ERF transcription factors.

## INTRODUCTION

Photosynthetic organisms started to emerge onto land approximately 500 million years ago in the Cambrian Period and then underwent further substantial evolutionary changes during the Ordovician Period (*1*). These early land plants, including relatives of extant bryophytes, were directly exposed to a gravitational acceleration of 1*g* on land, which was higher than the gravity they experienced in water due to the loss of buoyancy (*2*). Adaptation to this increase in effective gravity caused marked changes in the anatomy of the early land plants, requiring them to construct supporting structural tissues to orient their shoots toward the light (*3*). For example, the components and structure of the cell wall changed throughout land plant evolution after the divergence of the aquatic charophytes and the bryophytes (*4*).

The thickening of the cell wall led to reduced CO_2_ diffusion from the atmosphere into chloroplasts, decreasing the rate of photosynthesis (*5*) and leading plants to develop compensatory anatomical features. For example, in angiosperms, ferns and bryophytes, leaf anatomical traits such as mesophyll cell wall thickness and chloroplast surface area (*S*_c_) determine CO_2_ diffusional conductance and thus control the photosynthetic rate (*5*–*7*). If gravity accelerated the development of the cell wall, then *S*_c_ might have increased to compensate; however, no anatomical analyses of this phenomenon have been performed to elucidate the response of photosynthetic processes to gravity in bryophytes or vascular plants.

We previously reported that hypergravity of 10*g* (10 times Earth’s gravity) unexpectedly increased the photosynthetic rate in the bryophyte *Physcomitrium patens*, although the anatomical and molecular mechanisms underlying this effect remained unknown (*8*). In contrast, another study showed that photosynthesis was unchanged in wheat (*Triticum aestivum*) leaves under low gravity (*9*) and decreased after exposure to strong hypergravity (500 to 2000*g*) (*10*). These results suggest that bryophytes use unknown mechanisms not present in vascular plants to adapt to altered gravity; alternatively, differences in the magnitude of hypergravity (10*g* in moss versus 500 to 2000*g* in wheat) might have led to the differing effects on photosynthesis. Our previous study also revealed that the moss *P. patens* produced significantly more gametophores at 10*g* than at 1*g*, which has a positive impact on the photosynthesis rate (*8*). Therefore, some of the genes and phytohormones that affect gametophore formation in *P. patens* (*11*, *12*) could possibly also be involved in the hypergravity response.

Among the phytohormones, auxin and cytokinin are essential for determining the fate of a gametophore’s apical stem cell, which is important for the transition to the onset of gametophore formation and subsequent morphogenesis. In *Arabidopsis thaliana*, auxin levels increase under hypergravity conditions (300*g*), which increases the expression of lignin biosynthesis–related genes (*13*). To date, however, the coordination of anatomical traits with photosynthesis and the molecular mechanisms underlying the response to gravity have not been studied in bryophytes or vascular plants. Therefore, our aims were (i) to examine the effects of various degrees of hypergravity on photosynthesis, CO_2_ diffusional conductance, and the associated anatomical traits of *P. patens* and (ii) to gain insight into the molecular mechanisms involved in the anatomical and photosynthetic changes induced by hypergravity.

In this study, we show that increased gravity (6 and 10*g*) enhances photosynthesis in *P. patens* by improving CO_2_ diffusion through increased gametophore number and chloroplast size. Transcriptome analysis revealed the up-regulation of moss species-specific APETALA2/ethylene-responsive factor (AP2/ERF) transcription factor (TF) genes, including *ISSUNBOSHI1* (*IBSH1*; *Pp3c1_32440*), which links gravity responses to enhanced photosynthesis. Overexpression of *IBSH1* phenocopied the hypergravity responses, whereas the expression of its dominant repressor suppressed these responses. These findings suggest that the expansion of AP2/ERF TFs may have played a key role in moss plant adaptation to terrestrial environments.

## RESULTS

### Hypergravity alters photosynthetic rate through anatomical and morphological traits

**Fig. 1.**
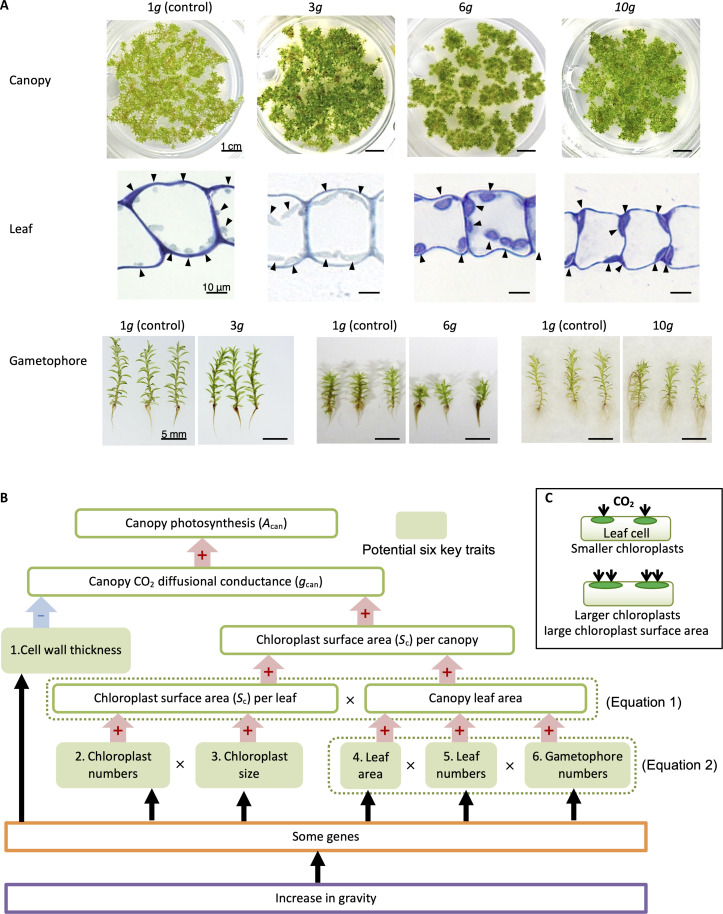
Images of *P. patens* and diagram of the six potential key traits influencing canopy-based photosynthesis. (**A**) Images of the populations of gametophores (canopy), individual gametophores, and light micrographs of leaf sections of *P. patens* grown under the control condition of 1*g* and hypergravity conditions of 3, 6, and 10*g*. Arrowheads in the images of the leaf section indicate chloroplasts. (**B**) Diagram of the six potential key traits influencing canopy-based photosynthesis (*A*_can_) in *P. patens* via canopy-based CO_2_ diffusional conductance (*g*_can_). An increase in gravity may induce changes in the expression of some genes that influence these six potential key traits. “+” and “–” indicate positive and negative effects, respectively. The relationships between the traits are shown in Eqs. 1 and 2. (**C**) Schematic illustrating the effect of chloroplast size on CO_2_ diffusion in the leaves of *P. patens.* Larger chloroplasts enhance CO_2_ diffusion into the leaves.

To investigate the response of *P. patens* to hypergravity, we grew populations of gametophores, known as “canopies” (*14*), under four gravity conditions (1, 3, 6, and 10*g*) for 8 weeks. Under the hypergravity conditions of 3, 6, and 10*g*, canopy growth appeared to be normal (Fig. 1A); however, gametophore stems tended to be shorter, and chloroplasts in the leaves were larger than in canopies grown at 1*g*.

Figure 1B illustrates a conceptual diagram of how canopy photosynthesis (*A*_can_) changes under hypergravity conditions owing to the alteration of traits through genetic regulation. Six anatomical or morphological traits—(i) cell wall thickness, (ii) chloroplast number, (iii) chloroplast size, (iv) leaf (phyllid) area, (v) leaf number, and (vi) gametophore number—are potential key traits that could be genetically regulated under hypergravity. The chloroplast number and size affect the *S*_c_ per leaf area, in which a larger chloroplast potentially increases the *S*_c_ for CO_2_ diffusion (Fig. 1C). Other traits, such as leaf area and number and gametophore number, increase the canopy leaf area. Increases in leaf *S*_c_ and canopy leaf area then increase canopy *S*_c_, enhancing the canopy CO_2_ diffusional conductance (*g*_can_) and lastly *A*_can_ (Eqs. 1 and 2).

**Fig. 2.**
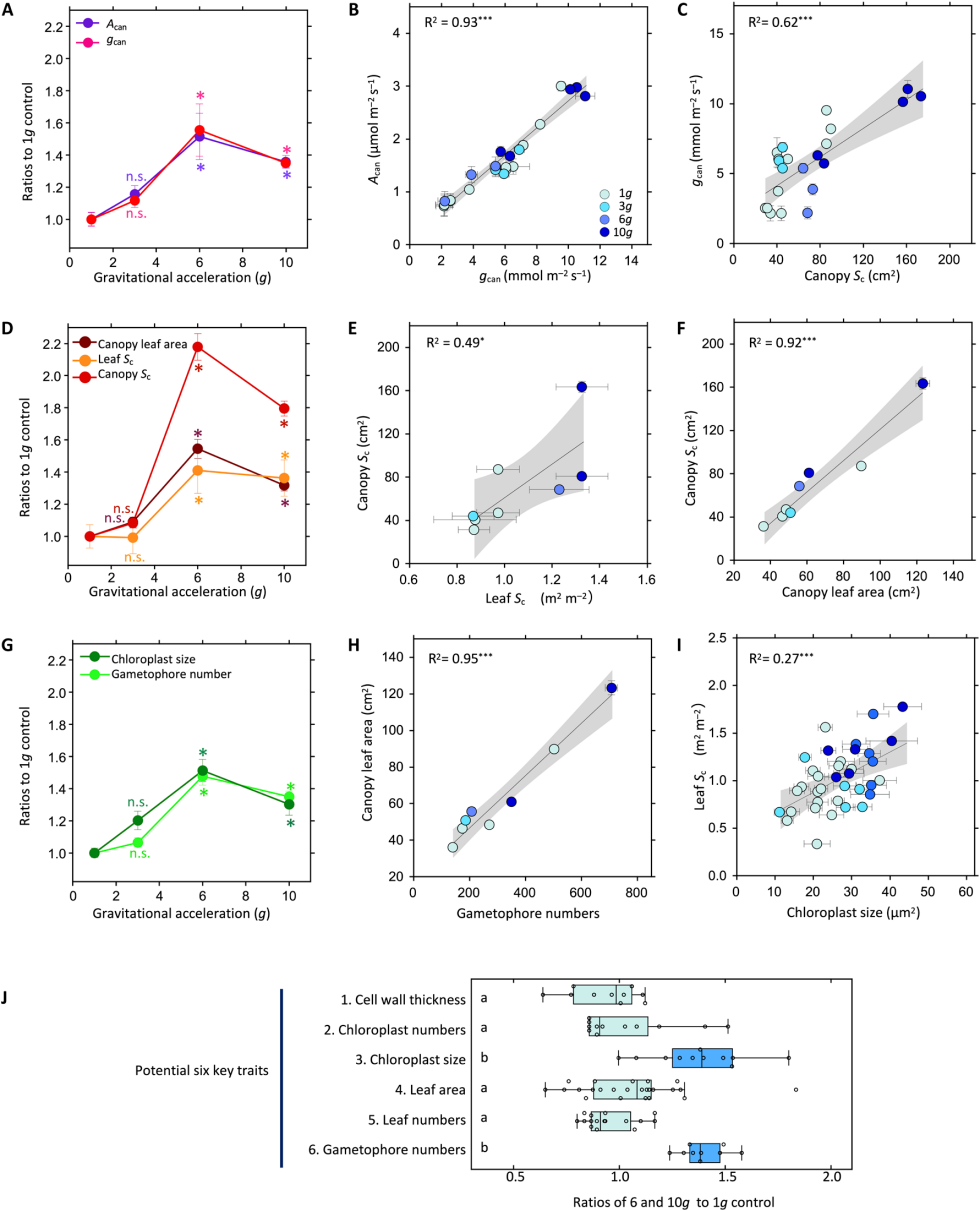
Photosynthetic, morphological, and anatomical traits of *P. patens* grown under hypergravity. (**A**) Ratios of seven *P. patens* traits under hypergravity of 3, 6, and 10*g* compared with the 1*g* controls. The traits were two canopy-level photosynthetic traits—(A) canopy photosynthesis (*A*_can_) and canopy CO_2_ diffusional conductance (*g*_can_) (*n* = 12 to 58; 3 to 12 canopies with repeated measurements); three morphological and anatomical traits—(**D**) canopy leaf area (*n* = 3 to 12), surface area of chloroplasts per leaf (*S*_c_) (*n* = 6 to 18), and canopy *S*_c_ (*n* = 3 to 12); and two potential key traits—(**G**) chloroplast size (*n* = 129 to 377) and gametophore number (*n* = 3 to 12). Bars indicate SEs. The differences between the 1*g* and hypergravity conditions were analyzed using analysis of variance (ANOVA), followed by Dunnett’s test (*P < 0.05; n.s., no significant difference, *P* > 0.05). (**B**, **C**, **E**, **F**, **H**, and **I**) Relationships between the seven traits. The regression coefficients are presented with significance levels of *P < 0.05 and ***P < 0.001. Gray areas show 95% confidence intervals. Bars indicate SEs. *R*^2^, coefficient of determination. (**J**) Box plots for the six potential key traits of *P. patens* (Fig. 1B), in which the ratios of pooled data for the hypergravity of 6 and 10*g* to 1*g* controls are shown. Shown are averaged values for each image of the leaf section for cell wall thickness (*n* = 10), chloroplast number (*n* = 12), and chloroplast size (*n* = 12); averaged values for each gametophore for leaf area and number (*n* = 17 to 24); and averaged values for each canopy for gametophore number (*n* = 9). Different letters indicate that the ratios of hypergravity to 1*g* controls were significantly different between traits, as tested by the Kruskal-Wallis test (*P* < 0.05).

First, gas exchange measurements for the moss canopies (Fig. 2A) showed that *A*_can_ was increased by 36 to 52% under 6 and 10*g*, whereas the increase was not significant at 3*g*. Concurrently, *g*_can_ was increased by 35 to 56% at 6 and 10*g* but not significantly changed at 3*g* (Fig. 2A). These results showed a strong positive correlation between *A*_can_ and *g*_can_ (Fig. 2B), suggesting that the enhancement in *A*_can_ under hypergravity conditions is primarily due to the enhancement of *g*_can_ in *P. patens*.

Next, we asked which traits are responsible for this increase in *g*_can_ under hypergravity. Two anatomical traits that could enhance CO_2_ diffusion in *P. patens* are (i) an increase in canopy *S*_c_ and (ii) a reduction in cell wall thickness (Fig. 1B). We found that the canopy *S*_c_ increased by 79 to 118% under hypergravity conditions of 6 and 10*g*, whereas the difference was not significant at 3*g* (Fig. 2D), suggesting that the increase in *S*_c_ per canopy contributes to the enhancement of CO_2_ diffusion under hypergravity; this was confirmed by a strong positive correlation between the canopy *S*_c_ and *g*_can_ (Fig. 2C). In contrast to canopy *S*_c_, we found no alterations in the cell wall thickness in leaves under any hypergravity condition (table S1).

We then explored the mechanisms underlying this increase in canopy *S*_c_ under hypergravity. The canopy *S*_c_ is a product of two traits, canopy leaf area and leaf *S*_c_ (Eqs. 1 and 2 and Fig. 1B). Both increased at 6 and 10*g* (Fig. 2D), resulting in strong positive correlations with canopy *S*_c_ (Fig. 2, E and F). Among the three traits that compose the canopy leaf area (Fig. 1B and Eq. 2), gametophore number increased at 6 and 10*g* (Fig. 2G), while leaf area and number per plant were unaltered (table S1), resulting in a strong positive correlation between canopy leaf area and gametophore number (Fig. 2H). This indicates that the enhanced gametophore proliferation under hypergravity increased the canopy *S*_c_ through an increase in the canopy leaf area.

Last, we investigated the main anatomical trait that increased leaf *S*_c_ under hypergravity. At 6 and 10*g*, the leaf chloroplast size increased, while again, there was no significant difference at 3*g* compared with at 1*g* (Fig. 2G). We counted the chloroplast number and found no significant changes under any of the hypergravity conditions (table S1). This indicates that the increase in leaf *S*_c_ was affected largely by the increase in chloroplast sizes, which is supported by the positive relationship between leaf *S*_c_ and chloroplast size (Fig. 2I). In summary, among the six anatomical and morphological traits potentially responsible for the response of *A*_can_ to hypergravity of 6 and 10*g* (Fig. 1B), we found that the two, chloroplast size and gametophore number, are the key traits (Fig. 2J) that enhance *g*_can_ (Fig. 2A). Notably, these key traits were not altered at 3*g*, revealing the existence of a threshold for the hypergravity response.

### Differentially expressed genes under hypergravity conditions

**Fig. 3.**
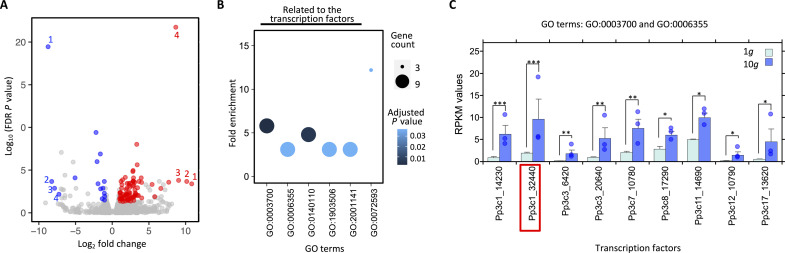
Gene expression in *P. patens* grown under hypergravity of 10*g*. (**A**) Volcano plot of DEGs in *P. patens* under hypergravity of 10*g* compared with a 1*g* control condition [*n* = 1292; false discovery rate (FDR) *P* < 1]. The 95 DEGs (table S3) are divided into 79 up-regulated DEGs (red) and 16 down-regulated DEGs (blue) under the hypergravity condition in comparison with the control (FDR *P* < 0.05). The top four most strongly up-regulated genes were *Pp3c2_24500*, *Pp3c14_10610*, *Pp3c20_20284*, and *Pp3c15_8300*, while the four most strongly down-regulated genes were *Pp3c17_931*, *Pp3c16_9850*, *Pp3c6_6030*, and *Pp3c10_5720*. (**B**) GO enrichment analysis. Fold enrichment, adjusted *P* values, and gene count are shown. The DEGs up-regulated at 10*g* were annotated as six GO terms, five of which were related to TFs. (**C**) Mean reads per kilobase per million reads (RPKM) values at 1 and 10*g* are shown for the nine genes, including eight encoding the AP2/ERF TFs. Bars show SEs (*n* = 3). FDR *P* values are shown as *P < 0.05, **P < 0.01, and ***P < 0.001.

To gain insight into the molecular mechanisms involved in these photosynthetic and anatomical responses, we conducted a transcriptome sequencing [RNA sequencing (RNA-seq)] analysis of hypergravity-treated *P. patens*. We identified 95 differentially expressed genes (DEGs) in the comparison between 1 and 10*g* in *P. patens* (Fig. 3A), of which 79 were up-regulated and 16 were down-regulated at 10*g*. We found that Gene Ontology (GO) terms related to TFs and reactive oxygen species processes were significantly enriched among the DEGs (Fig. 3B and table S2). In addition, 18 genes appeared to be specific to *P. patens* and of unknown function (table S3). We further examined the TFs in the 95 DEGs and found that nine TFs were up-regulated by hypergravity (Fig. 3C and table S3). Moreover, to our surprise, eight of the nine TFs belong to the same AP2/ERF TF family (Fig. 3C and table S3).

### Uncharacterized AP2/ERF TFs are enriched among hypergravity-related DEGs

The AP2/ERF family in *A. thaliana* and *P. patens* consists of four major subfamilies: AP2, related to ABSCISIC ACID INSENSITIVE3 (ABI3)/VIVIPAROUS1(VP1) (RAV), ERF, dehydration responsive element binding protein (DREB), and an outlier group. The eight AP2/ERF TFs that we identified in the DEGs contain only one AP2 domain and no B3 DNA binding domain. A protein BLAST (BLASTp) search against the Arabidopsis Information Resource (TAIR) database revealed that the eight AP2/ERF TFs showed the highest similarity to members of the ERF or DREB subfamily; however, this high similarity was limited to the AP2 domains, with relatively low similarity in other regions (fig. S1 and table S4). We further identified the AP2 domain–containing proteins by a BLASTp search using each of the eight selected AP2-domain *P. patens* protein sequences as a query, in other mosses, such as *Bryum argenteum*, *Sphagnum fallax*, and *Sphagnum magellanicum*; in hornworts, such as *Anthoceros punctatus* and *Anthoceros agrestis*; in liverworts, such as *Marchantia polymorpha*; in Zygnematophyceae green algae, as the closest algal relatives of land plants, such as *Spirogloea muscicola*, *Mesotaenium endlicherianum*, and *Zygnema circumcarinatum*; in lycophyte, such as *Selaginella moellendorffii*; and in flowering plants, in addition to *A. thaliana*, such as *Oryza sativa*. We then constructed a phylogenetic tree among the AP2 domains of *P. patens*, *B. argenteum*, *S. fallax*, *S. magellanicum*, *A. punctatus*, *A. agrestis*, *M. polymorpha*, *S. muscicola*, *M. endlicherianum*, *Z. circumcarinatum*, *S. moellendorffii*, *A. thaliana*, and *O. sativa*. Notably, all eight AP2/ERFs of *P. patens* grouped exclusively with AP2/ERFs from other mosses within a distinct clade (named cluster 2 herein), separate from the DREB, ERF, RAV, and AP2 subfamilies (fig. S2A). This clustering suggests that these genes were uniquely amplified in mosses and may perform specialized functions in this lineage. Multiple sequence alignment revealed that a negatively charged amino acid insertion (Asp/Glu) at position 11 is predominantly present in the moss (*P. patens*, *B. argenteum*, *S. fallax*, and *S. magellanicum*) AP2/ERFs from cluster 2 (fig. S2B). A similar insertion was not found in *A. punctatus*, *A. agrestis*, *M. polymorpha*, *Z. circumcarinatum*, *S. moellendorffii*, *A. thaliana*, or *O. sativa*, implying that this insertion may reflect unique DNA binding properties or affinity for the regulation of target gene expression.

Hypergravity is an abiotic stress, and many AP2/ERF TFs are known to play important roles in abiotic stress responses. We therefore investigated whether the expression patterns of the eight hypergravity-responsive AP2/ERF TFs were similar to those of two well-known stress-responsive AP2/ERF TFs, Pp3c3_31490 for the DREB subfamily and Pp3c21_16540 for the ERF subfamily (*15*). To our surprise, we noted contrasting genetic responses (fig. S2C). Dehydration, heat, and cold stresses induced no increases but rather decreased the expression of the eight AP2/ERF TFs, whereas these stresses overall increased the expression of the two stress-responsive TFs. By contrast, under different light conditions (dark, red, blue, ultraviolet B, or far-red), the changes in the expression of the eight AP2/ERF TFs were more notable than those in the stress-responsive TFs. Furthermore, the expression of the two stress-responsive TFs did not change at 10*g*.

### Hypergravity increases AP2/ERF TF protein accumulation in protonema and gametophore

**Fig. 4.**
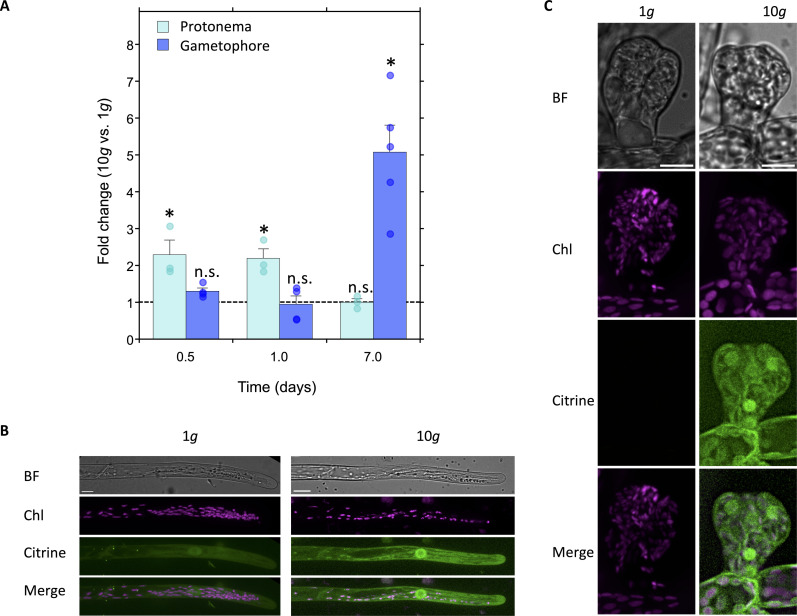
Expression and localization of Pp3c1_32440 TF. (**A**) Fold change (10*g* versus 1*g*) of *Pp3c1_32440* TF in protonemal cells and gametophores measured by qRT-PCR analysis at three distinct time points. Expression levels were normalized to 1*g*, which was set to 1. Ubiquitin-conjugating enzyme E2 was used as the reference gene for normalization. Data represent means of fold change, with bars showing SEs (*n* = 3 to 5). The differences between 1 and 10*g* were analyzed using Welch’s *t* test (*P < 0.05 and n.s.). (**B** and **C**) Z-stack projection images showing the localization of the Pp3c1_32440: Citrine fusion protein in 5-day-old protonemal cells (B) and gametophore buds (C) under 1 and 10*g* conditions. Confocal images of protonemal cells were captured after 1 day of 10*g* culture, and images of buds were taken after 7 days of 10*g* culture. Citrine fluorescence (Citrine, green) indicates Pp3c1_32440 localization, and chlorophyll autofluorescence (Chl, magenta) marks chloroplasts. BF indicates bright field images. The observed localization pattern was consistent in apical protonemal cells (*n* = 32) and buds (*n* = 25 at the two to four cell stages under 1 and 10*g* culture). Scale bars, 10 μm.

On the basis of our RNA-seq analysis, we identified eight AP2/ERF TFs that were significantly induced under hypergravity culture. To validate the RNA-seq results, we performed quantitative reverse transcription polymerase chain reaction (qRT-PCR) analysis of these eight AP2/ERF TFs in gametophyte plants cultured at 1 and 10*g* for 2 months. Among them, the gene *Pp3c1_32440* was selected for further analysis due to its high fold-change induction (fig. S3A). According to the *P. patens* electronic Fluorescent Pictograph (eFP) browser, *Pp3c1_32440* is predominantly expressed in protonemal tissues, rhizoids, and mature sporophytes with comparatively weaker expression observed in gametophores (fig. S3B). To examine the spatiotemporal expression patterns of *Pp3c1_32440* under normal growth and hypergravity conditions, we quantified its expression levels in two distinct tissue types: protonemal tissues and gametophore tissues, including rhizoids (Fig. 4A). The analysis was performed at three time points: 0.5, 1, and 7 days after shifting to 10*g* culture. The results showed that in protonemal tissues, *Pp3c1_32440* expression was up-regulated approximately twofold at 0.5 and 1 days post–10*g* stimulation, with no significant change observed in gametophore tissues during this shorter time frame. However, after 7 days of 10*g* culture, the expression level of *Pp3c1_32440* increased by more than 4.5-fold in the gametophore including rhizoid tissues, while remaining unchanged in protonemal tissues. These results suggest that hypergravity effectively activates the transcriptional expression of *Pp3c1_32440* across a range of tissue types. Notably, the filamentous stage, protonema, responds more rapidly to hypergravity, whereas the gametophore stage including rhizoids exhibits a pronounced response only after prolonged hypergravity exposure.

To further understand the spatiotemporal regulation of Pp3c1_32440 TF at the protein level, we generated fluorescent protein knock-in lines (Pp3c1_32440: Citrine #8, #10, and #11), where the citrine fluorescent protein sequence was fused to the C terminus of Pp3c1_32440 TF. Under normal 1*g* growth conditions, all transgenic lines exhibited dual localization of the TF in both the cytosol and nucleus of protonemal cells (Fig. 4B for line #10 and fig. S4C for lines #8 and #11)*.* To examine whether Pp3c1_32440 TF protein levels increase under hypergravity (10*g*) conditions, we cultured the Pp3c1_32440: Citrine #10 line and analyzed fluorescence intensity across different tissues. After 1 day of culture at 10*g*, protonemal cells exhibited stronger fluorescence intensity in both the cytosol and nucleus compared to cells grown under 1*g* conditions (Fig. 4B). Furthermore, while buds (early gametophores) displayed very weak or undetectable signals under 1*g* conditions, consistent with the transcriptional profile of the AP2/ERF TF, fluorescence intensity in both the cytosol and nucleus increased in buds after 1 week of 10*g* culture (Fig. 4C), suggesting protein accumulation in response to prolonged 10*g* stimulation. These findings indicate that the localization of the AP2/ERF TF protein is regulated both spatially and temporally, complementing its transcriptional regulation. Notably, Pp3c1_32440 TF accumulation is enhanced under hypergravity conditions in protonema, and during the early stages of gametophytic development, where the TF is typically absent under normal growth conditions.

### Overexpressing an *AP2/ERF* TF phenocopies the responses of 10*g*-grown moss

We next investigated whether the overexpression of the *AP2/ERF* TFs at normal 1*g* conditions might increase photosynthesis or exhibit any phenocopy of *P. patens* grown under hypergravity conditions. We thus generated four independent transgenic lines overexpressing *Pp3c_32440*, Ox#13, Ox#28, Ox#75, and Ox#202, confirming the overexpression of the transgene using qRT-PCR (fig. S3C).

**Fig. 5.**
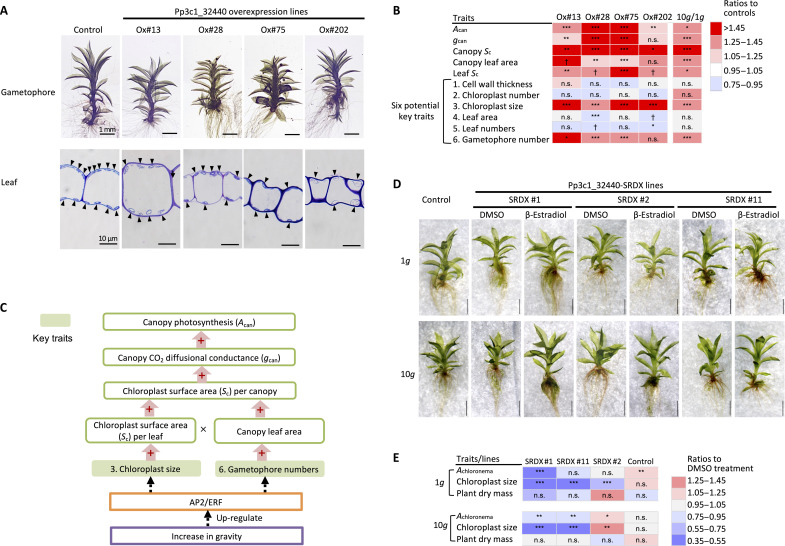
Phenotypes of *Pp3c1_32440* (*IBSH1*)–overexpressing plants and dominant repressor plants, and the hypothetical diagram leading to hypergravity responses that are mediated via IBSH1 and its related AP2/ERFs. (**A**) Light micrographs of gametophores and sections of the leaves of the controls and the four *Pp3c1_32440*-overexpressing lines of *P. patens*. Arrowheads in the images of the leaf section indicate chloroplasts. (**B**) Ratios of the photosynthetic (*n* = 12 to 18), morphological (*n* = 6 to 30), and anatomical traits (*n* = 6 to 40; *n* = 242 to 316 for chloroplast size) of the *Pp3c1_32440*-overexpressing lines to the wild-type *P. patens* (control), and ratios of the wild-type *P. patens* (control) grown under 10*g* compared to those of wild-type plants grown under 1*g*. Differences between overexpressing lines and control were analyzed by ANOVA, followed by Dunnett’s test (†*P* < 0.1, *P < 0.05, **P < 0.01, ***P < 0.001, and n.s.). The data for 10*g* are duplicated in Fig. 2 (A, D, and G). (**C**) Diagram of the photosynthetic response of *P. patens* to hypergravity. AP2/ERF TFs are up-regulated in response to hypergravity and increase chloroplast size and gametophore growth, resulting in enhanced photosynthesis. + indicates a positive effect. (**D**) Light microscopy images of gametophores from wild-type control plants and three independent *Pp3c1_32440-SRDX* transgenic lines (#1, #2, and #11) after 1 month of growth under 1 and 10*g* conditions. Scale bars, 1 mm. (**E**) Photosynthesis (*n* = 9), chloroplast size (*n* = 33 to 49), and plant dry mass (*n* = 3) were measured in the chloronema of *Pp3c1_32440-SRDX* lines and wild type of *P. patens* grown under 1 and 10*g* conditions. Ratios of β-estradiol–treated plants to dimethyl sulfoxide (DMSO)–treated controls are represented by color-coded bars. Differences between β-estradiol– and DMSO-treated plants were assessed using Welch’s *t* test (*P < 0.05, **P < 0.01, ***P < 0.001, and n.s.).

We then examined the traits of all *P. patens* overexpression lines. The lines overexpressing *Pp3c_32440* mimicked the key changes seen at 10*g* (Fig. 5, A and B). When compared with the wild-type plants grown at 1*g* (control), the shoots of the gametophores were shorter, and the chloroplasts were larger in the overexpression lines (Fig. 5A), which was similar to the responses obtained under hypergravity (Fig. 1A). All overexpression lines had a 22 to 70% increase in *A*_can_ when compared with the controls (Fig. 5B and table S5). As might be expected from the response found in the 6*g*- or 10*g*-cultured wild-type plants, the three overexpression lines (Ox#13, Ox#28, and Ox#75) that showed high *A*_can_ also showed a significant increase in *g*_can_ (17 to 108% higher than that of the controls). In addition, all the overexpression lines exhibited significant increases in canopy *S*_c_ (44 to 111% greater) because of the increased canopy leaf area (by 8 to 17%) and leaf *S*_c_ (by 31 to 73%). Although the numbers of chloroplasts and leaves were overall not significantly different from those for control plants (table S5), the two key traits that enhance CO_2_ diffusion under hypergravity conditions, gametophore number and chloroplast size (Fig. 2J), were increased overall in the *AP2/ERF* overexpression lines (by 30 to 60% and 29 to 88%, respectively; Fig. 5B and table S5). Thus, we concluded that the overexpression of this single *AP2/ERF* mimicked the hypergravity responses of the physiology and anatomy of *P. patens* by increasing the chloroplast size and gametophore number, which consequently enhanced CO_2_ diffusion and thus increased photosynthetic activity at the canopy level (Fig. 5C).

### Inhibition of Pp3c1_32440 TF function suppresses hypergravity-induced changes

Overexpression of the *Pp3c1_32440* TF phenocopied hypergravity responses in the moss (Fig. 5, A and B). Consequently, we sought to determine whether the responses induced by 10*g* require the transcriptional activation of target genes associated with AP2/ERF TFs. To this end, we generated a transgenic plant overexpressing a dominant repressor of *Pp3c1_32440* TF by creating a chimeric protein fused with the dominant SUPERMAN REPRESSION DOMAIN X (SRDX) repressor domain, referred to as *Pp3c1_32440-SRDX*. Three distinct transgenic lines exhibiting inducible overexpression of the dominant suppressor, designated as lines #1, #2, and #11, were used to examine alterations in moss physiology. To confirm the successful overexpression of *Pp3c1_32440-SRDX* in these lines, we performed qRT-PCR. The analysis revealed that lines #1 and #11 exhibited the highest induction levels of *Pp3c1_32440-SRDX*, while line #2 displayed comparatively milder induction (fig. S5), suggesting that the function of Pp3c1_32440 was effectively suppressed in β-estradiol–treated lines #1 and #11.

Subsequently, various traits, including photosynthesis, chloroplast size, plant dry mass, and shoot length under different gravity conditions, were analyzed to evaluate the effects of disrupting the normal regulation of Pp3c1_32440 downstream target genes (Fig. 5, D and E, and table S6, A and B). As expected, under 10*g* conditions, β-estradiol–treated *Pp3c1_32440-SRDX*–overexpressing lines (#1 and #11) exhibited decreases in photosynthesis (*A*_chloronema_) and chloroplast size compared to the dimethyl sulfoxide (DMSO) mock-treated plants (Fig. 5E). Notably, decreases in photosynthesis and chloroplast size were also observed in lines #1 and #11 under 1*g* conditions, suggesting that Pp3c1_32440 may play a role in regulating these traits even under normal 1*g* gravity conditions. Regarding shoot length, hypergravity conditions (3, 6, and 10*g*) significantly reduced gametophore shoot length compared to 1*g*, and overexpression of *Pp3c1_32440* under 1*g* conditions led to decreased shoot length compared to wild-type plants (table S6A). However, under 10*g* conditions, β-estradiol–treated *Pp3c1_32440-SRDX*–overexpressing lines exhibited significantly longer shoot lengths than the mock-treated plants [table S6A; DMSO versus β-estradiol (EST)], suggesting that *Pp3c1_32440-SRDX*–overexpressing plants are less susceptible to hypergravity-induced shoot growth inhibition.

Together, our findings suggest that the 10*g*-induced response is at least partially mediated by a member of the AP2/ERF TF family, with Pp3c1_32440 likely required for the transcriptional activation of its downstream targets.

## DISCUSSION

We first showed that the hypergravity-induced increase in the photosynthesis rate (*A*_can_) in *P. patens*, at 6 and 10*g*, but not 3*g*, was attributable to changes in CO_2_ diffusional conductance (*g*_can_). A strong dependence of canopy-based photosynthesis on *g*_can_ was previously reported for 26 mosses and seven liverworts (*7*); however, the factors that induce this increase in *g*_can_ were poorly understood. Here, we uncovered key traits that determined *g*_can_ in response to hypergravity, namely, chloroplast size and gametophore number. Further, changes in these key traits were reproduced by one of the eight *AP2/ERF* TFs, *Pp3c1_32440*, that were uniquely amplified in the *P. patens* genome; the overexpression of *Pp3c1_32440* also phenocopied changes in many other traits observed under hypergravity, while suppression of Pp3c1_32440 inhibited hypergravity responses. Therefore, on the basis of the overexpression phenotype, we named this gene *ISSUNBOSHI1* (*IBSH1*), inspired by a Japanese fairy tale about an inch-high, strong boy.

To our knowledge, there has been no study about how hypergravity alters chloroplast size, one of the two key traits. Previous studies at 1*g* have reported that greater numbers of smaller chloroplasts effectively increased the *S*_c_ for CO_2_ diffusion in angiosperms (*5*, *16*). Here, we identified an increase in chloroplast size and no change in chloroplast number for *P. patens* grown under hypergravity, as well as *IBSH1* overexpression lines under 1*g*. Several studies have revealed that diverse environmental factors, such as drought, high temperature, and cold stress, have impacts on both chloroplast size and number in angiosperms (*17*–*20*). For example, some deciduous trees enhance their photosynthesis by increasing their chloroplast size as a light acclimation strategy (*21*). Furthermore, we previously reported that an AP2/ERF TF of the ERF subfamily (Pp3c17_10170 in fig. S2A; Cluster1) regulates salt-induced chloroplast division in *P. patens* with no notable change in chloroplast size (*22*). Thus, the regulation of chloroplast size and number to adapt photosynthesis to environmental changes may be more common in land plants than previously thought and can be dynamically tuned under different environmental conditions and in different tissues and plant species. At present, however, because the molecular mechanisms that regulate the balance between chloroplast number and size are complex and little studied, how *S*_c_ is regulated for CO_2_ diffusion is poorly understood (*5*–*7*, *23*, *24*). Thus, the eight AP2/ERFs that we identified here could be useful probes for elucidating these processes.

How is another key trait, gametophore number, regulated by the AP2/ERF TFs? Several studies have reported the regulatory genes and phytohormones that affect gametophore formation in *P. patens* (*11*, *12*), among which auxin and cytokinin are particularly essential. However, we found no DEGs involved in either the auxin or cytokinin pathways in *P. patens*, although the auxin level has been found to be elevated under hypergravity conditions (300*g*) in *A. thaliana* (*13*). Furthermore, in our DEG list, we did not identify any genes involved in gametophore formation (*11*, *25*, *26*); for example, DEFECTIVE KERNEL1 (DEK1) and NO GAMETOPHORES 1 (NOG1) are necessary for gametophytic apical stem cell formation and are regulated by AP2/ERF TFs, called AINTEGUMENTA, PLETHORA and BABY BOOM (APBs) (*27*). Although these APBs regulate gametophore formation, they bear two AP2 domains and so belong to a different subfamily from the eight AP2/ERFs that we found here (*27*). In contrast, a recent study reported that the overexpression in *P. patens* of *PpMACRO2*, a likely fungal-derived gene with an unknown function, increased the number and shortened the height of the gametophore (*28*). More notably, among the up-regulated genes in the *PpMACRO2*-overexpressing plants, the same three *AP2/ERF* TFs (*Pp3c3_6420V3.1*, *Pp3c1_14230V3.1*, and *Pp3c7_10780V3.1*) were identified as in the present study, suggesting that the gene regulatory networks for gametophore formation driven by PpMACRO2 and hypergravity may partially overlap, which likely results in the similar phenotypes in both *PpMACRO2*- and *IBSH1*-overexpressing plants.

Our analysis suggests that a notable moss lineage-specific gene amplification of AP2/ERF paralogs, including the eight hypergravity-responsive AP2/ERFs, may have occurred (fig. S2A). This further prompted us to infer the canonical functions of these paralogs, especially focusing on the eight AP2/ERFs. Because the magnitude of gravity has been one of the most stable environmental factors over Earth’s long history, remaining constant at 1*g* (*29*), it is very unlikely that responsiveness to hypergravity is the inherent function of the eight AP2/ERF TFs. Then, we performed expression analyses using the *Physcomitrella* eFP browser, indicating that most of the eight *AP2/ERF* TFs are expressed in protonemata, gametophores, and rhizoids (fig. S3B), suggestive of broad function in these tissues. We also found that the expression of the eight *AP2/ERF* TFs varies with light levels and quality (fig. S2C); thus, we speculate that the canonical function of the eight AP2/ERF TFs might be to regulate the adaptive response to changes under light conditions (*30*). Therefore, it will be interesting to elucidate how these TFs, which originally responded to light, acquired responsiveness to changes in gravity magnitude and regulate photosynthesis activity by dissecting cis elements in the promoter region of the eight *AP2/ERF* genes, e.g., *IBSH1*, and elucidating the target genes.

Although we previously reported hypergravity-induced increases in growth and photosynthesis (*8*), the link between gravity, growth, photosynthesis, and genetic control has remained unclear. To our knowledge, we provide the first comprehensive elucidation of the mechanism by which hypergravity promotes growth via enhanced photosynthesis, integrating genetic, cellular, individual, and canopy-level responses in moss plants. We identified an important TF, IBSH1, that mediates the hypergravity response. Many AP2/ERF TFs in angiosperms remain uncharacterized, raising the possibility that some AP2/ERF TFs with functions similar to IBSH1 may exist in vascular plants or other groups of bryophytes. Identifying such AP2/ERF TFs or expressing *IBSH1* in angiosperms could lead to the development of plants with enhanced photosynthetic capacity and increased biomass production. Moreover, with growing interest in long-term human activities in space (*31*–*34*), understanding plant responses to diverse gravitational environments is an emerging frontier (*35*, *36*). Our findings not only advance fundamental knowledge of plant adaptation to gravity but also have important implications for future space agriculture, contributing to sustainable life support systems for humanity beyond Earth.

## MATERIALS AND METHODS

### Plant material and cultivation under hypergravity conditions

A bryophyte species, *Physcomitrium* (formerly known as *Physcomitrella*) *patens* (Hedw.) Mitt., was used for all experiments. The cultivation of *P. patens* was performed from 2014 to 2016 at the laboratory of the University of Toyama, Toyama, Japan, as described previously (*8*). Briefly, gametophores of *P. patens* that were grown in a growth chamber at 25°C under continuous white light were cut into pieces 3 mm in length from the shoot apex. Twelve segments were planted and grown for 35 days on 50 ml of BCD medium (*37*) in six plant boxes 7.5 cm in diameter and 10.3 cm in height (Biochemical Science, Tokyo, Japan). The samples were then subjected to three levels of hypergravity—1*g* (control), 3*g*, and 6 or 10*g*—with the 10*g* treatment and its control repeated twice. The experiments were performed using a custom-built centrifuge equipped with a light-emitting diode (LED) emitting a photosynthetic photon flux density (PPFD) of 67 μmol m^−2^ s^−1^ (MK3, Matsukura, Kurobe, Japan) and grown at 25°C for 8 weeks, with the addition of half-strength BCD medium once a week to prevent complete desiccation. This device enabled long-term plant cultivation with irradiance under controlled hypergravity conditions. The CO_2_ concentration in the plant boxes was confirmed to be similar between the hypergravity and 1*g* environments, and the vibration experienced during the hypergravity treatment was so small that it imposed no effect on the growth of *P. patens* (*38*). For the 1*g* controls, six plant boxes were placed in the same temperature-controlled room and grown for 8 weeks under the same LEDs with the same PPFD as those used for the hypergravity experiments.

At the end of the experiments, *P. patens* formed dense populations of gametophores in the plant boxes. Photosynthesis was measured for the populations of shoots, e.g., the “canopy,” after removing the rhizoids and agar medium. Morphological and anatomical traits that were potentially related to the canopy photosynthetic functions and/or canopy growth were measured for 4 to 12 randomly selected gametophores collected from two or three plant boxes. The number of gametophores collected for these analyses was <1% of that of the entire population in a single plant box, so this sample collection did not affect the canopy traits. Transporting the plants from the University of Toyama to the Kyoto Institute of Technology, Kyoto, Japan, took a few days, during which the plant samples were stored at 4°C. Upon arrival, the plants were placed at room temperature (25°C) for 1 day before the photosynthesis measurements.

### Measurement of *g*_can_ using the carbon isotope method

Photosynthesis measurements for the canopy of *P. patens* at a fully turgid mass were performed using a custom-made gas exchange system constructed at the Kyoto Institute of Technology (*8*, *39*) (see Supplementary Text and fig. S6). The rhizoids and agar medium were removed from the samples, after which the whole canopy of *P. patens* in the two plant boxes was used for the measurement of *A*_can_. The projected canopy area was measured using a scanner. The canopy temperature was adjusted to 26°C, the flow rate was 200 to 350 ml min^−1^, the relative humidity was ~90%, and the CO_2_ concentration was set at 400 μmol mol^−1^. A red and blue LED (red:blue = 8:1; LEDRB-630DL, Opto Code Co., Tokyo, Japan) was used as a light source. Light-response curves were generated using a previously described model (*40*), confirming that the canopy photosynthesis of *P. patens* was light saturated at a PPFD of 200 μmol m^−2^ s^−1^, with no reduction in photosynthesis occurring at a PPFD of 500 μmol m^−2^ s^−1^ (*8*). The photosynthesis rate was therefore measured at a PPFD of 400 μmol m^−2^ s^−1^ to determine the light-saturated photosynthesis of the canopy of *P. patens*. To measure the *g*_can_ of *P. patens*, gas exchange and carbon isotope discrimination were measured concurrently using a custom-designed system (see Supplementary Text). The canopy photosynthesis was measured before and after CO_2_ collection, with three repeated measurements of the canopy in each plant box.

Few studies have examined canopy-based photosynthesis activity in bryophytes. Centrifugal equipment for hypergravity experiments was developed for the present study, so the validity of the data generated was verified by cross-checking the data generated at 1*g* with those from previous studies. Canopy-based photosynthetic traits and leaf traits are summarized in table S7. The values were within the range of previous studies, in which the reproducibility of some of the values obtained in the previous study was confirmed using different centrifugal equipment (*8*).

### Measurement of canopy and leaf traits

The number of plants in a canopy was determined by counting the number of gametophores in a plant box (*n* = 3 to 6). To determine the leaf numbers and leaf area, 4 to 12 average-sized fresh gametophores from two to three plant boxes were photographed (PowerShot SX210 IS, Canon, Tokyo, Japan), and the leaf numbers were counted. To obtain the leaf area, 12 images of average-sized leaves were taken using a light microscope (BX51-33, Olympus, Tokyo, Japan) and then analyzed using ImageJ software (*41*).

### Measurement of morphological and anatomical traits

For the anatomical measurements of leaves, light micrographs were taken using a microscope (BX51-33, Olympus). The images were digitally recorded with a charge-coupled device camera (DP22, Olympus) and analyzed using ImageJ (*41*). The leaves were obtained at 3 to 5 mm from the base of the stems of three to five average-sized gametophores. They were fixed in 2.5% (v/v) glutaraldehyde in 0.2 M sodium phosphate buffer (pH 7.4) for 20 min, postfixed in 1% (w/v) OsO_4_ solution at 4°C for 3 hours in darkness, and then embedded in Spurr’s resin (Low Viscosity Resin kit, TAAB Laboratories Equipment, Aldermaston, UK). Transverse sections of stems and leaves (1 μm thick) were stained with 1% (w/v) toluidine blue solution.

The numbers of all chloroplasts per cell were counted for five cells from the tip of the leaves for six transverse sections, observed at ×1000 magnification. At the same time, the size of all these chloroplasts was measured by tracing the images of the chloroplasts using ImageJ (*41*). The cell wall thicknesses in the leaves were measured for the third to seventh cell from the margin of the lamina of four gametophores, with two parts of a cell wall attached to the chloroplasts being selected for the measurements (*n* = 40) using ×1000-magnification light micrographs.

The *S*_c_ facing the air per leaf area (leaf *S*_c_) was estimated using the method described previously (*6*, *42*), with modifications. The chloroplasts were assumed to be oblate spheroids, for which the dimensional ratio (major axis/minor axis) was estimated to be 2.0 based on preliminary observations (*n* = 160). The curvature factor was assumed to be 1.14, according to a previous study (*43*). Leaf *S*_c_ was measured at ×1000 magnification at the central part of the lamina in six leaf sections for all chloroplasts attached to the cell walls that were facing the air.

### Integrated analysis of *g*_can_ and anatomical or morphological traits

Leaf *S*_c_ is one of the determinant factors for the CO_2_ diffusional conductance of plants (*5*); therefore, for the CO_2_ diffusional conductance of the *P. patens* canopy (*g*_can_), the canopy-based surface area of chloroplasts (canopy *S*_c_), e.g., the sum of the *S*_c_ for the canopy of *P. patens*, is the decisive factor. Canopy *S*_c_ is related to anatomical and morphological factors, such as

$$\text{Canopy}\;S_{c}=\text{leaf}\;S_{c}\times \text{canopy leaf area}$$ (1)

$$\begin{array}{c} \begin{array}{c} \text{Canopy leaf area}=\text{average leaf area}\\ \;\times \text{leaf numbers}\times \text{gametophore numbers} \end{array} \end{array}$$ (2)

Leaf *S*_c_ is affected by both chloroplast number and chloroplast size and reaches a maximum with increasing numbers of small chloroplasts (*5*). If the number of chloroplasts is unchanged, then large chloroplasts potentially increase the surface area for CO_2_ diffusion if the shape of the chloroplasts, e.g., the ratio of thickness and width of the chloroplasts, is unchanged.

### Measurement of photosynthesis and anatomical traits in *Pp3c1_32440-SRDX* lines

Photosynthesis measurements for the chloronema of *P. patens* at a fully turgid mass were performed as described in the previous section. The agar medium was removed from the samples, after which the chloronema of *P. patens* on the film was used for the measurement of photosynthesis (*A*_chloronema_). The projected chloronema area was measured using a scanner. The chloronema temperature was adjusted to 26°C, the flow rate was 400 ml min^−1^, the relative humidity was ~90%, and the CO_2_ concentration was set at 400 μmol mol^−1^. After the photosynthesis measurement, chloronema of *P. patens* was dried at 60°C for 24 hours and then measured its dry weight. The size of all chloroplasts per cell was counted for second cells from the tip of the chloronema, observed at ×400 magnification, and measured by tracing the images of the chloroplasts using ImageJ (*41*).

### RNA-seq analysis

For RNA-seq, gametophores grown under 10*g* for 8 weeks were immersed in RNAlater RNA stabilization reagent (QIAGEN, Hilden, Germany), and the total RNA was purified using an RNeasy Plant Mini kit (QIAGEN) according to the manufacturer’s protocol. The obtained RNA was frozen in liquid nitrogen and stored at −80°C. mRNA-seq libraries were created using the Illumina TruSeq RNA sample prep kit (Illumina, San Diego, CA, USA) according to the manufacturer’s standard protocol. Samples were then subjected to single-end sequencing of 100–base pair reads on an Illumina NextSeq platform. Reads passing Illumina’s chastity filtering process were selected for further analysis. The single reads are available from the DNA Data Bank of Japan (DDBJ) Sequence Read Archive under accession number DRA008190 [DRR172632 to DRR172637].

### Plasmid construction of AP2/ERF *Pp3c1_32440*

The full-length coding sequence of *Pp3c1_32440* was PCR amplified from first-strand cDNA using PrimeSTAR Max DNA polymerase (Takara Bio) with the primers #901 and #902. The PCR product was inserted into *Sal*I- and *Eco*RV-linearized pENTR1A vector (Thermo Fisher Scientific, Waltham, MA, USA) using the hot fusion method (*44*). For constitutive overexpression of *Pp3c1_32440*, the sequence from the entry vector was transferred via a Gateway LR reaction (Thermo Fisher Scientific) into the pT1OG vector containing the *PpEF1* promoter (*27*). To generate an inducible dominant suppressor of *Pp3c1_32440*, the coding sequence without the stop codon was PCR amplified and cloned into the entry vector. This sequence was then subcloned into pGX8DR via a Gateway LR reaction, resulting in the plasmid pPGX8-Pp3c1_32440-SRDX.

For the in-fame knock-in construct of Pp3c1_32440: Citrine fusion protein, DNA fragments (~1.5 kb) upstream and downstream of the stop codon were PCR amplified using the primer sets #1215, #1216, #1217, and #1218, respectively. The upstream fragment was cloned into *Sal*I- and *Eco*RV-linearized pCTRN-NPTII vector (GenBank: AB697058), ensuring that the reading frame of *Pp3c1_32440* was in frame with citrine. The downstream fragment was then inserted into the *Sma*I-*Xba*I sites, positioned downstream of the selective marker cassette in pCTRN-NPTII.

### Transformation and confirmation of transgenic plants

All plasmid constructs were introduced into moss protoplasts using polyethylene glycol–mediated transformation, as previously described (*37*). Before transformation, the DNA plasmids were linearized with the appropriate restriction enzymes. Transgenic lines were selected twice on BCDAT agar medium supplemented with antibiotics corresponding to the selection markers encoded by the vectors.

To validate the Pp3c1_32440: Citrine knock-in lines, genomic DNA (gDNA) was extracted from colonies that survived antibiotic selection. PCR amplification was performed on both the flanking regions and the regions encompassing the introduced genetic construct to confirm its integration into the moss genome. Specifically, primer combinations #1372 (#Pcmv-R) and #2256 were used for the 5′ upstream homologous recombination (HR) region, while #621 (#NptII-F1) and #2257 were used for the 3′ downstream HR region. The resulting PCR products were analyzed by agarose gel electrophoresis, and colonies displaying the expected bands were selected for further investigation (fig. S4, A and B).

For overexpression lines, a total of 304 of the transgenic lines, selected on BCDAT agar medium containing Zeocin as a selection marker, were transferred to BCD agar medium to promote gametophore production. Among these, four lines (Ox#13, Ox#28, Ox#75, and Ox#202) exhibiting distinct phenotypic differences were selected for further analysis. To confirm the overexpression of *Pp3c1_32440*, protonemal tissue from these four lines was cultured on BCDAT agar medium for 5 days. The expression level of *Pp3c1_32440* was quantified by qRT-PCR with the primer sets #579 and #580, as detailed below. Each sample was analyzed in three technical replicates, with measurements performed on three independent biological samples.

For dominant suppressor lines, a total of eight transgenic lines, selected on BCDAT agar medium containing hygromycin as a selection marker, were transferred to BCD agar medium and cultured for 2 weeks to induce gametophore production. The lines were then treated with 1 μM β-estradiol or 0.098% DMSO as a control. Among these, three lines (#1, #2, and #11), which exhibited distinct phenotypic differences between the DMSO and β-estradiol treatments, were selected for further analysis. To confirm the overexpression of the *Pp3c1_32440-SRDX* sequence, protonemal tissues from three independent lines (#1, #2, and #11) were cultured on BCDAT agar medium for 5 days, followed by treatment with either 1 μM β-estradiol or 0.098% DMSO as a control for 1 day. The expression level of *Pp3c1_32440-SRDX* was quantified by qRT-PCR with the primer sets #579 and #580, as detailed below. Each sample was analyzed in three technical replicates, with measurements performed on three independent biological samples.

### Quantitative RT-PCR

To validate the RNA-seq analysis of *Pp3c1_32440* gene expression, 8-week-old gametophores cultured under either 10 or 1*g* conditions were analyzed (fig. S3C). For spatiotemporal gene expression analysis of *Pp3c1_32440* (Fig. 4A), wild-type protonemal tissue (5 days old) and gametophores, including rhizoids, were cultured under 10*g* conditions, with 1*g* serving as a control, at three distinct time points: 7, 12, and 24 days. Total RNA was extracted using the RNeasy Plant Mini kit (QIAGEN) according to the manufacturer’s instructions. The cDNA synthesis was performed using ReverTra Ace qPCR RT Master Mix with gDNA Remover (Toyobo, Osaka, Japan). qRT-PCR was performed on Applied Biosystems 7300 Real-Time PCR System (Thermo Fisher Scientific) with Power SYBR Green PCR Master Mix (Thermo Fisher Scientific). Relative fold changes in gene expression were calculated using the formula 2^–ΔC(t)^, where ΔC(t) = C(t)_gene of interest_ – C(t)_ub-conjugating E2_, with normalization to the expression level of the ubiquitin-conjugating enzyme E2 (*Pp3c14_21480v3.1*). Each qRT-PCR experiment was performed with at least three technical replicates and three biological replicates. Primer sequences used in this study are listed in table S8.

### Growth conditions for the overexpression and dominant suppressor lines

The same genotype of *P. patens* as that used for the hypergravity experiment was used as the wild type. Sixteen segments of gametophores of the wild type and four overexpressing lines, Ox#13, Ox#28, Ox#75, and Ox#202, were planted in BCDATG or BCDAT agar medium in petri dishes 9 cm in diameter and 1.5 cm in height (Biochemical Science). They were grown under constant white light conditions (~50 μmol m^−2^ s^−1^) provided by 40-W white fluorescent light bulbs (FLR40S-EX-N/M/36, Mitsubishi-Osram, Kanagawa, Japan) at 22° to 25°C at Hokkaido University, Sapporo, Japan, for 2 months, to generate gametophores.

For dominant suppressor lines, three independent *Pp3c1_32440-SRDX* lines (#1, #2, and #11) were cultured on BCD agar medium in petri dishes under constant white light conditions (~25 μmol m^−2^ s^−1^) at 25°C for 2 weeks to generate gametophores or chloronemata. These gametophores or chloronemata were then treated with either 1 μM β-estradiol to induce the expression of the *Pp3c1_32440* gene containing the SRDX domain sequence or 0.098% DMSO as a mock control. Following treatment, the gametophores or chloronemata were subjected to a 10*g* gravitational force or maintained at 1*g* as a control for 1 month (gametophores) or 4 days (chloronemata), respectively.

### Bioinformatics and data analyses

As the reference genome of *P. patens*, Phytozome v13.0 was used for the bioinformatics analysis. CLC Genomics Workbench software (QIAGEN) was used to map the reads to the genome sequence of *P. patens* for the calculation of the reads per kilobase per million reads (RPKM) values and the comparison of the RPKM values among samples. The obtained *P* values were corrected on the basis of the Benjamini-Hochberg method (*45*). DEGs satisfying the thresholds of adjusted *P* < 0.05 and fold change > 2 or < 0.5 were subjected to a GO enrichment analysis using ShinyGO version 0.76. The gene names and their assigned GO terms were based on Phytozome v13.0.

### Multiple alignment and construction of the phylogenetic tree

To construct the phylogenetic tree of AP2 domain–containing proteins, 27 *P. patens*, 9 *B. argenteum*, 6 *S. fallax*, 6 *S. magellanicum*, 4 *A. punctatus*, 4 *A. agrestis*, 13 *M. polymorpha*, 5 *S. muscicola*, 5 *M. endlicherianum*, 16 *Z. circumcarinatum*, 8 *S. moellendorffii*, 25 *A. thaliana*, and 17 *O. sativa* sequences were used. The *P. patens* protein sequences also included the eight selected AP2 domain proteins found to be differentially expressed under hypergravity (Pp3c3_6420V3.1, Pp3c3_20640V3.1, Pp3c12_10790V3.1, Pp3c17_13620V3.1, Pp3c1_32440V3.1, Pp3c1_14230V3.1, Pp3c7_10780V3.1, and Pp3c11_14690V3.1).

To search for homologous sequences in *A. thaliana*, *P. patens*, *B. argenteum*, *S. fallax*, *S. magellanicum*, *A. punctatus*, *A. agrestis*, *M. polymorpha*, *S. muscicola*, *M. endlicherianum*, *Z. circumcarinatum*, *S. moellendorffii*, and *O. sativa*, each of the eight selected AP2-domain *P. patens* protein sequences (full protein sequences and AP2 domain–only sequences) was used, in turn, as a query to perform a BLASTp search [databases for BLAST search: https://phytozome-next.jgi.doe.gov/ (V3.3) for *P. patens*; https://figshare.com/articles/dataset/Bryum_argenteum_genome_assembly/21226328 (V7.0) for *B. argenteum*; https://phytozome-next.jgi.doe.gov/ (V1.1) for *S. fallax* and *S. magellanicum*; www.hornworts.uzh.ch/en/hornwort-genomes.html (V1.0) for *A. punctatus* and *A. agrestis*; https://marchantia.info/ (V7.1) for *M. polymorpha*; https://mycocosm.jgi.doe.gov/Spimu1_1/Spimu1_1.home.html (V1.0) for *S. muscicola*; https://phycocosm.jgi.doe.gov/Mesen1/Mesen1.home.html (SAG 12.97) for *M. endlicherianum*; https://mycocosm.jgi.doe.gov/Zygcir6981b_1/Zygcir6981b_1.home.html (SAG 698-1b), https://mycocosm.jgi.doe.gov/Zygcir1560_1/Zygcir1560_1.home.html (UTEX 1560), https://mycocosm.jgi.doe.gov/Zygcir1559_1/Zygcir1559_1.home.html (UTEX 1559), and https://mycocosm.jgi.doe.gov/Zygcyl6981a_1/Zygcyl6981a_1.home.html (SAG 698-1a_XF) for *Z. circumcarinatum*; https://phytozome-next.jgi.doe.gov/ (V1.0) for *S. moellendorffii*; www.arabidopsis.org/ (TAIR10) for *A. thaliana*; https://phytozome-next.jgi.doe.gov/ (V7.0) for *O. sativa*]. According to the results of the BLAST analysis, the first five candidate homologs from *P. patens*, *B. argenteum*, *S. fallax*, *S. magellanicum*, *A. punctatus*, *A. agrestis*, *M. polymorpha*, *S. muscicola*, *M. endlicherianum*, *Z. circumcarinatum*, *S. moellendorffii*, *A. thaliana*, or *O. sativa* with the lowest *E* values were subjected to further alignment analysis. In cases where the same candidates were identified after BLAST search using the full protein or domain-only protein sequence, the duplications were removed. In addition, from the BLAST list for *P. patens*, additional proteins from subfamilies DREB and ERF were randomly selected (nine additional proteins in total). There are many proteins in the DREB or ERF subfamilies, so these additional proteins were chosen on the basis of having the lowest E values.

MEGA11 software (*46*) was used for the protein alignment and phylogenetic tree construction. The alignment of protein sequences was carried out using the multiple sequence comparison by log-expectation (MUSCLE) method with the following parameters: gap penalty (gap open: −2.90; gap extend: 0.00; hydrophobicity multiplier: 1.20); cluster method unweighted pair group method with arithmetic mean (UPGMA). After the sequence alignment, any gap regions were manually removed. An AP2 domain with a length of 55 or 56 amino acids was isolated. The AP2 domain was determined according to the conserved sequence described in a previous study (*47*). To construct the phylogenetic tree, AP2 domain sequences without any gaps were used (the length of the domain after removing the insertion is 55 amino acids). The evolutionary tree was inferred using the maximum likelihood method and Jones-Taylor-Thornton (JTT) matrix–based model (*48*). The bootstrap method (500 replications) was used as a phylogenetic test.

### Confocal microscopy

Pp3c1_32440-citrine lines were observed with an inverted microscope (ECLIPSE Ti2, Nikon) equipped with a spinning disk head (X-Light V3, CrestOptics) with a 1.4 numerical aperture 60× oil immersion objective (Plan Apo VC 60× Oil, Nikon) at room temperature. Illumination with 520-nm laser light was used for citrine excitation, and emission filters were 560/40 nm. Illumination with 640-nm laser light was used for chlorophyll autofluorescence, and emission filters were 660/735 nm. Image acquisition was controlled by MetaMorph (Molecular Devices). All images were processed using ImageJ.

### Statistical analysis

The effect of hypergravity (3, 6, or 10*g*) on the traits of *P. patens* was tested using analysis of variance (ANOVA) and Dunnett’s multiple comparison test, using 1*g* as the control. Similarly, the differences in the traits between AP2/ERF overexpression lines and controls were tested using Dunnett’s multiple comparison test. For the regression analysis between the traits obtained from the hypergravity experiment, the data were fitted by a linear regression (*y* = *ax* + *b*), for which the significance of the regression was tested using the *F* test. For the potential six key traits, namely, cell wall thickness, chloroplast number, chloroplast size, leaf area, leaf number, and gametophore number, the ratios of the values for 6 and 10*g* to the 1*g* control are shown in box plots, with the difference between traits tested using the Kruskal-Wallis test (*P* < 0.05). In the experiments of *Pp3c1_32440-SRDX* lines, the effect of β-estradiol was tested using Welch’s *t* test. The effects of treatments and gravity were tested using two-way ANOVA. We performed statistical analysis using R version 4.4.1 and R Commander Version 2.9-2.

## Supplementary Materials

### The PDF file includes:

Supplementary Text Figs. S1 to S6 Tables S1 to S8 Legends for data S1 to S7 References
