# Supplementary material for: First contact with greater gravity: Moss plants adapted via enhanced photosynthesis mediated by AP2/ERF transcription factors
Source: Sci Adv. 2025 Jul 16;11(29):eado8664. doi: 10.1126/sciadv.ado8664 (PMC12266102; doi:10.1126/sciadv.ado8664)
Supplement: Supplementary file 1 — Supplementary Text Figs. S1 to S6 Tables S1 to S8 Legends for data S1 to S7 References [file sciadv.ado8664_sm.pdf]

Science Advances
AAAS

Supplementary Materials for

First contact with greater gravity: Moss plants adapted via enhanced photosynthesis mediated by AP2/ERF transcription factors

Yuko T. Hanba *et al.*

Corresponding author: Yuko T. Hanba, hanba@kit.ac.jp; Tomomichi Fujita, tfujita@sci.hokudai.ac.jp

Sci. Adv. **11**, eado8664 (2025)
DOI: 10.1126/sciadv.ado8664

The PDF file includes:

Supplementary Text
Figs. S1 to S6
Tables S1 to S8
Legends for data S1 to S7
References

Other Supplementary Material for this manuscript includes the following:

Data S1 to S7

Supplementary Text

Additional methods for photosynthetic measurements

To quantify the photosynthesis rate, rhizoids were removed from the gametophores, which were then completely immersed in distilled water to ensure their saturation. Excess external capillary water was removed from the gametophore surface using filter paper, giving a fully turgid mass (TM). The TM of the gametophores was generally close to the optimal tissue water content for photosynthesis (*49*). To confirm this, the photosynthesis measurements were first performed under saturated irradiance (500 μmol m^{-2} s^{-1}) for a drying cycle of gametophores (4–5 hours; Fig. S6). Fully turgid gametophores were subsequently rewetted to restore the surface liquid layer, and the fresh mass was determined before measurement. The photosynthesis rate was then successively measured, with the fresh mass measured at intervals of 30 min. Photosynthesis measurement continued until the fresh mass was nearly constant. After the measurements were finished, the samples were dried to a constant weight at 60°C and used to obtain the dry mass. Tissue water content was expressed as the percentage of water content at a given time as related to the water content at full turgor (*49*):

$$\text{Tissue water content (\%)} = \frac{\text{Fresh mass} - \text{Dry mass}}{\text{Turgid mass} - \text{Dry mass}} \times 100 \qquad \text{(S1)}$$

During the photosynthesis measurements, the gametophores were placed in a custom-made resin chamber (10 cm × 10 cm × 4 cm), with the temperature monitored using a thermocouple. Boundary layer conductance was measured in accordance with area by obtaining a calibration curve using filter papers with different areas (data not shown). Two fans placed inside the chamber mixed the air completely. The response of the net CO_2 assimilation rate to changing irradiance was obtained at seven photosynthetic photon flux densities (PPFD) from 0 to 800 μmol m^{-2} s^{-1} at TM. At each PPFD, at least 5 min was required for the photosynthesis rate to stabilize.

Additional methods for determining the CO_2 diffusional conductance using the isotope method

The CO_2 diffusional conductance of a canopy of *P. patens* (g_{can}) was estimated using an isotope method. The g_{can} of *P. patens* was measured at TM because, like the photosynthesis rate, the bryophyte g_{can} is close to optimum at TM (*50*). A custom-made gas exchange system with a plant chamber was connected to a vacuum line for CO_2 collection, and the collected CO_2 was subjected to an isotope analysis using a dual-inlet stable isotope mass spectrometer (Delta XP; Thermo Fisher Scientific, Waltham, MA, USA). The observed carbon isotope discrimination during photosynthesis was calculated using the following equation (*51*):

$$\Delta_o\ (‰) = \frac{1000 \times \xi\left(\delta^{13}C_a - \delta^{13}C_{ref}\right)}{1000 + \delta^{13}C_a - \xi\left(\delta^{13}C_a - \delta^{13}C_{ref}\right)} \qquad \text{(S2)}$$

where $\delta^{13}C_a$ and $\delta^{13}C_{ref}$ are the carbon isotopic compositions in the chamber and in reference air, respectively. $\xi = C_{ref}/(C_{ref} - C_a)$, where ξ is the ratio of CO_2 entering into the chamber to the net CO_2 fixation by the plant canopy, and C_a and C_{ref} are the CO_2 concentrations in the leaf chamber and in reference air, respectively. ξ was kept at approximately 20 during the measurements.

The g_{can} was affected by an external layer of liquid water on the surface of the gametophores of *P. patens*. Assuming optimal hydration, i.e., the gametophores are fully hydrated without additional water on their surface (*49*) (Tissue water content ≈ 100%), g_{can} is calculated using the equations reported in a previous study (*52*):

$$g_{can} = \frac{1+t}{1-t}\left(b - a_i - \frac{eR_d}{(A+R_d)}\right)\frac{A}{C_a} \Big/ (\Delta_i - \Delta_o - \Delta_e - \Delta_f) \quad \text{(S3)}$$

$$t = \frac{(1+a_b)\,E}{2g_{ac}^{t}} \quad \text{(S4)}$$

where a_b is a fractionation factor through the boundary layer, E is the transpiration rate and g^t_{ac} is the total conductance to CO_2 diffusion. b (30‰) is the fractionation associated with Rubisco carboxylation (*52*), a_i (1.8‰) is the fractionation factor for dissolution and diffusion through water and R_d is day respiration. The parameter e, which is associated with day respiration, was calculated as

$$e = \delta^{13}C_{tank} - \delta^{13}C_{atmosphere} \quad \text{(S5)}$$

assuming no fractionation by day respiration (*51*, *53*). $\delta^{13}C_{tank}$ ranged from −30‰ to −35‰, and $\delta^{13}C_{atmosphere}$ was assumed to be −8‰. The fractionation occurred when $C_s = C_c$ and without respiratory fractionation is:

$$\Delta_i = \frac{1}{(1-t)}a_b + \frac{1}{(1-t)}\left((1+t)\,b - a_b\right)\frac{C_s}{C_a} \quad \text{(S6)}$$

where C_s is the CO_2 concentration of the surface of the gametophores or chloronema of *P. patens*. The fractionation with respiration was calculated as:

$$\Delta_e = \frac{1+t}{1-t}\left(\frac{e\,R_d}{(A+R_d)\,C_a}(C_s - \Gamma^*)\right) \quad \text{(S7)}$$

where Γ^* is the CO_2 compensation point in the absence of R_d. C^*, the apparent CO_2 compensation point (*54*), was used as a proxy of Γ^* (*55*). A C^* of 45 µmol mol^{-1} and an R_d of 0.01 µmol m^{-2} s^{-1} was calculated, as described in a previous study (*52*). All g_{can} measurements were performed at TM.

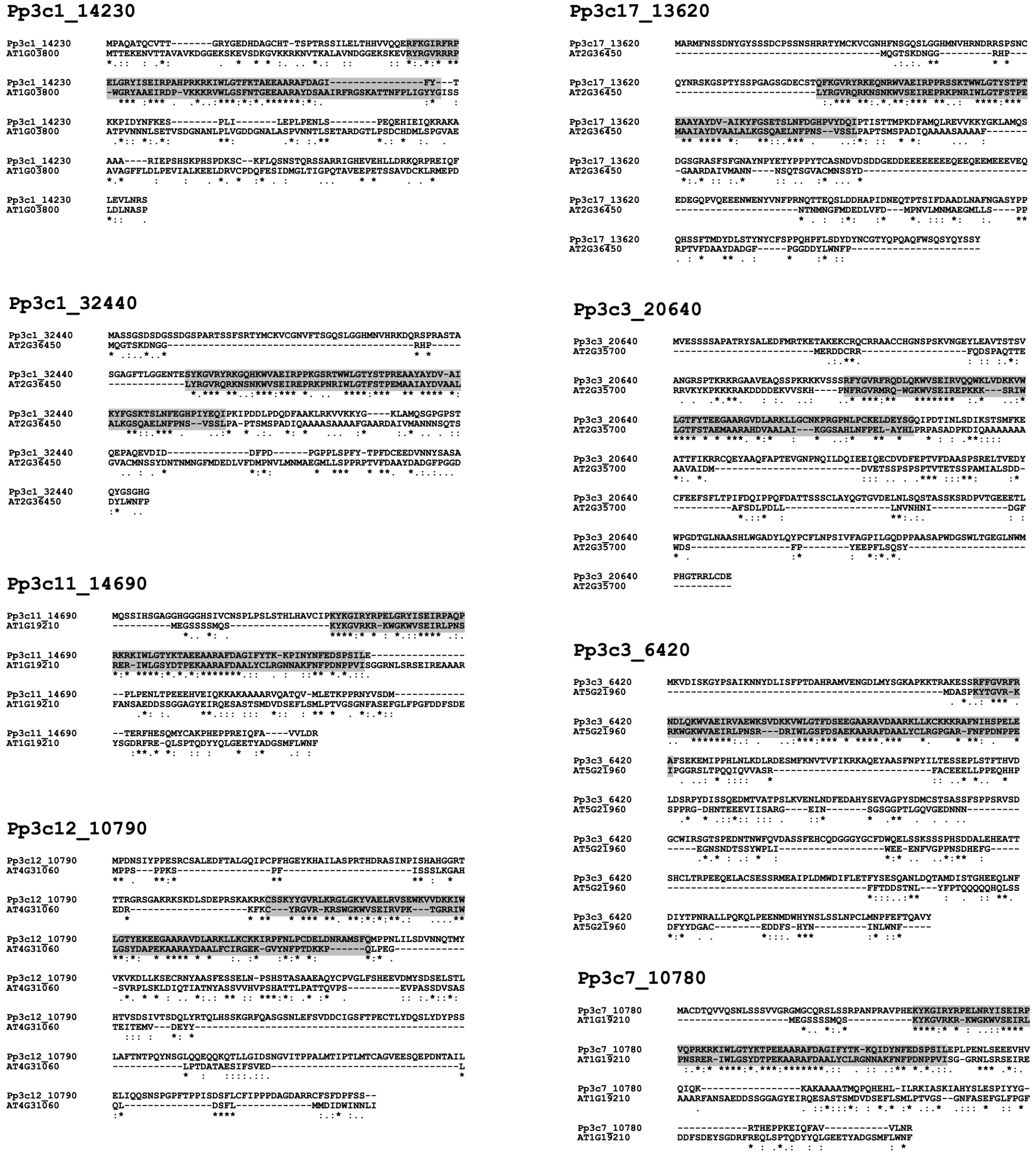

Fig. S1. The eight AP2/ERF transcription factors in *Physcomitrium patens* aligned with the top hits in *Arabidopsis thaliana*.

The eight APETALA2/ETHYLENE-RESPONSIVE FACTOR (AP2/ERF) proteins upregulated at 10*g* in *Physcomitrium patens* aligned with the top-hit protein sequences from *Arabidopsis thaliana.* The *A. thaliana* sequences were obtained by a BLASTP search using the whole amino acid sequence of the Physcomitrium protein as a query. The AP2 domain is shaded in gray. * indicates identical amino acid residues; “ : ” and “ . ” indicate conservation between groups of strongly and weakly similar properties, respectively.

A

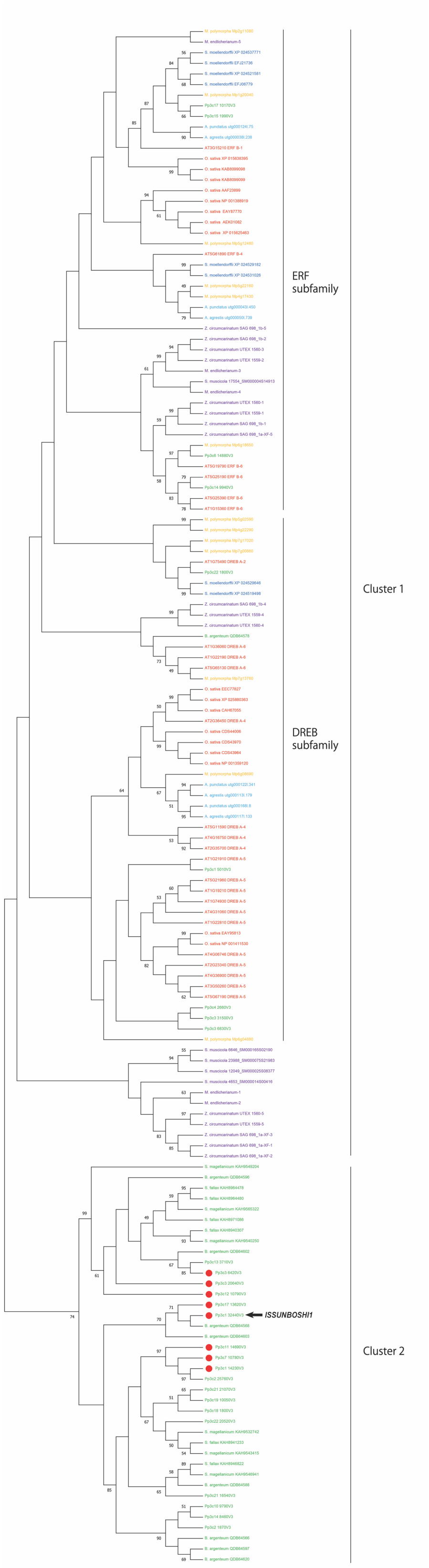
ERF subfamily
DREB subfamily
Cluster 1
Cluster 2
ISSUNBOSHI1

B

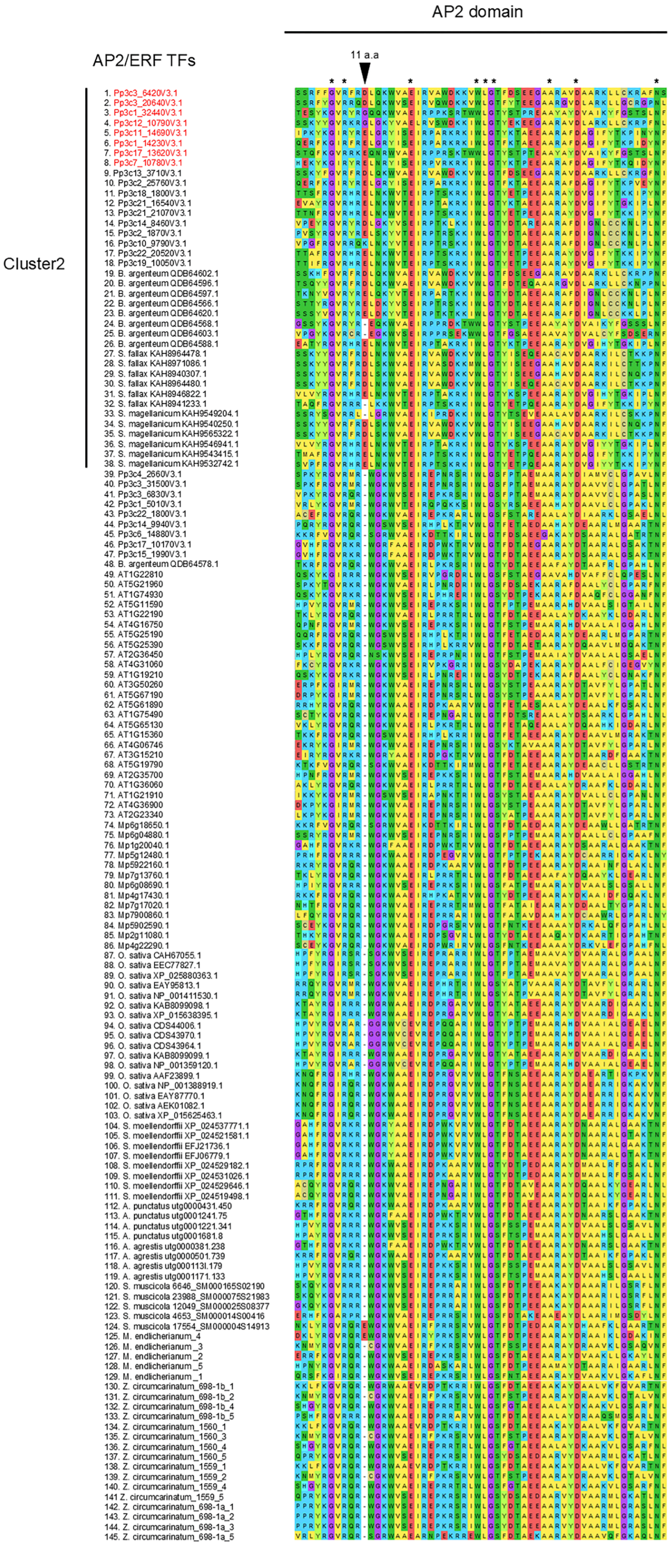
AP2 domain
AP2/ERF TFs
11 a.a
Cluster2
1. Pp3c3_6420V3.1
2. Pp3c3_20640V3.1
3. Pp3c1_32440V3.1
4. Pp3c12_10790V3.1
5. Pp3c11_14690V3.1
6. Pp3c1_14230V3.1
7. Pp3c17_13620V3.1
8. Pp3c7_10780V3.1
9. Pp3c13_3710V3.1
10. Pp3c2_25760V3.1
11. Pp3c18_1800V3.1
12. Pp3c21_16540V3.1
13. Pp3c21_21070V3.1
14. Pp3c14_8460V3.1
15. Pp3c2_1870V3.1
16. Pp3c10_9790V3.1
17. Pp3c22_20520V3.1
18. Pp3c19_10050V3.1
19. B. argenteum QDB64602.1
20. B. argenteum QDB64596.1
21. B. argenteum QDB64597.1
22. B. argenteum QDB64566.1
23. B. argenteum QDB64620.1
24. B. argenteum QDB64568.1
25. B. argenteum QDB64603.1
26. B. argenteum QDB64588.1
27. S. fallax KAH8964478.1
28. S. fallax KAH8971086.1
29. S. fallax KAH8940307.1
30. S. fallax KAH8964480.1
31. S. fallax KAH8946822.1
32. S. fallax KAH8941233.1
33. S. magellanicum KAH9549204.1
34. S. magellanicum KAH9540250.1
35. S. magellanicum KAH9565322.1
36. S. magellanicum KAH9546941.1
37. S. magellanicum KAH9543415.1
38. S. magellanicum KAH9532742.1
39. Pp3c4_2660V3.1
40. Pp3c3_31500V3.1
41. Pp3c3_6830V3.1
42. Pp3c1_5010V3.1
43. Pp3c22_1800V3.1
44. Pp3c14_9940V3.1
45. Pp3c6_14880V3.1
46. Pp3c17_10170V3.1
47. Pp3c15_1990V3.1
48. B. argenteum QDB64578.1
49. AT1G22810
50. AT5G21960
51. AT1G74930
52. AT5G11590
53. AT1G22190
54. AT4G16750
55. AT5G25190
56. AT5G25390
57. AT2G36450
58. AT4G31060
59. AT1G19210
60. AT3G50260
61. AT5G67190
62. AT5G61890
63. AT1G75490
64. AT5G65130
65. AT1G15360
66. AT4G06746
67. AT3G15210
68. AT5G19790
69. AT2G35700
70. AT1G36060
71. AT1G21910
72. AT4G36900
73. AT2G23340
74. Mp6g18650.1
75. Mp6g04880.1
76. Mp1g20040.1
77. Mp5g12480.1
78. Mp5922160.1
79. Mp7g13760.1
80. Mp6g08690.1
81. Mp4g17430.1
82. Mp7g17020.1
83. Mp7900860.1
84. Mp5902590.1
85. Mp2g11080.1
86. Mp4g22290.1
87. O. sativa CAH67055.1
88. O. sativa EEC77827.1
89. O. sativa XP_025880363.1
90. O. sativa EAY95813.1
91. O. sativa NP_001411530.1
92. O. sativa KAB8099098.1
93. O. sativa XP_015638395.1
94. O. sativa CDS44006.1
95. O. sativa CDS43970.1
96. O. sativa CDS43964.1
97. O. sativa KAB8099099.1
98. O. sativa NP_001359120.1
99. O. sativa AAF23899.1
100. O. sativa NP_001388919.1
101. O. sativa EAY87770.1
102. O. sativa AEK01082.1
103. O. sativa XP_015625463.1
104. S. moellendorffii XP_024537771.1
105. S. moellendorffii XP_024521581.1
106. S. moellendorffii EFJ21736.1
107. S. moellendorffii EFJ06779.1
108. S. moellendorffii XP_024529182.1
109. S. moellendorffii XP_024531026.1
110. S. moellendorffii XP_024529646.1
111. S. moellendorffii XP_024519498.1
112. A. punctatus utg0000431.450
113. A. punctatus utg0001241.75
114. A. punctatus utg0001221.341
115. A. punctatus utg0001681.8
116. A. agrestis utg0000381.238
117. A. agrestis utg0000501.739
118. A. agrestis utg0001131.179
119. A. agrestis utg0001171.133
120. S. muscicola 6646_SM000165S02190
121. S. muscicola 23988_SM000075S21983
122. S. muscicola 12049_SM000025S08377
123. S. muscicola 4653_SM000014S00416
124. S. muscicola 17554_SM000004S14913
125. M. endlicherianum_4
126. M. endlicherianum_3
127. M. endlicherianum_2
128. M. endlicherianum_5
129. M. endlicherianum_1
130. Z. circumcarinatum_698-1b_1
131. Z. circumcarinatum_698-1b_2
132. Z. circumcarinatum_698-1b_4
133. Z. circumcarinatum_698-1b_5
134. Z. circumcarinatum_1560_1
135. Z. circumcarinatum_1560_3
136. Z. circumcarinatum_1560_4
137. Z. circumcarinatum_1560_5
138. Z. circumcarinatum_1559_1
139. Z. circumcarinatum_1559_2
140. Z. circumcarinatum_1559_4
141 Z. circumcarinatum_1559_5
142. Z. circumcarinatum_698-1a_1
143. Z. circumcarinatum_698-1a_2
144. Z. circumcarinatum_698-1a_3
145. Z. circumcarinatum_698-1a_5

C

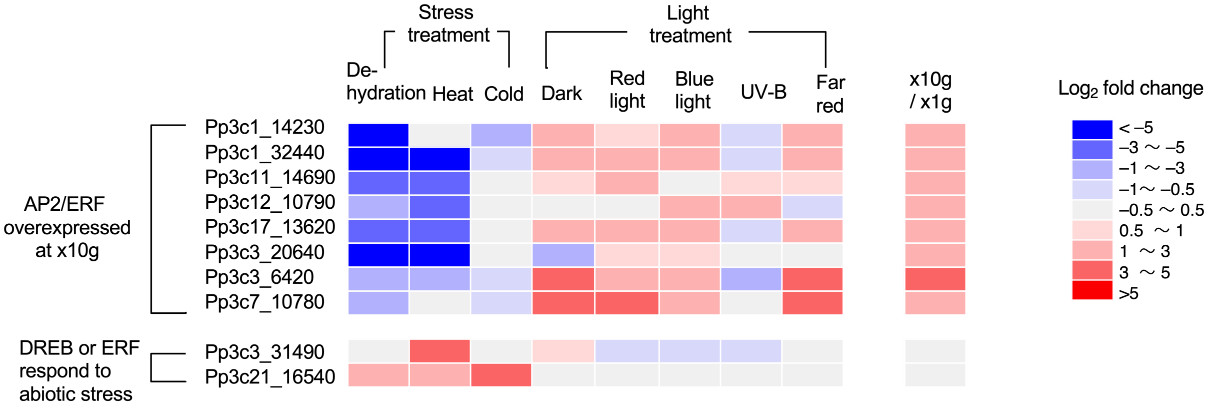

Fig. S2. Characteristic features of the eight AP2/ERF transcription factors.

(A) A phylogenetic tree of APETALA2/ETHYLENE-RESPONSIVE FACTOR (AP2/ERF) constructed across multiple plant lineages, including mosses (*Physcomitrium patens*, *Bryum argenteum*, *Sphagnum fallax*, *Sphagnum magellanicum*; highlighted in green), hornworts (*Anthoceros punctatus*, *Anthoceros agrestis*; highlighted in light blue); liverwort (*Marchantia polymorpha*; highlighted in orange), Zygnematophyceae green algae (*Spirogloea muscicola*, *Mesotaenium endlicherianum*, *Zygnema circumcarinatum*; highlighted in purple), lycophyte (*Selaginella moellendorffii*; highlighted in dark blue), and angiosperms (*Arabidopsis thaliana*, *Oryza sativa*; highlighted in red). Two major subfamilies, the ERF and the dehydration responsive element binding (DREB) proteins, are shown. The eight *P. patens* AP2/ERF TFs identified in this study as being differentially expressed under hypergravity are marked by red circles. Bootstrap values greater than 50 are shown at the nodes. **(B)** Multiple alignment of AP2 domain–containing protein sequences from 27 *P. patens*, 9 *B. argenteum*, 6 *S. fallax*, 6 *S. magellanicum*, 4 *A. punctatus*, 4 *A. agrestis*, 13 *M. polymorpha*, 5 *S. muscicola*, 5 *M. endlicherianum*, 16 *Z. circumcarinatum*, 8 *S. moellendorffii*, 25 *A. thaliana* and 17 *O. sativa*. A negatively charged amino acid insertion (Asp/Glu) at the 11th amino acid position of the AP2 domain (indicated by an arrowhead) appears to be unique to some moss AP2/ERF TFs (*P. patens*, *B. argenteum*, *S. fallax*, *S. magellanicum*), including the eight TFs differentially expressed under hypergravity in this study. Asterisks at the top line indicate conserved amino acids among all sequences. Amino acid residues are shown as a single letter code, with identical residues shown in the same color. Note that the gap region at the position of the 11th amino acid was removed when the phylogenetic tree was constructed in (**A**). **(C)** The gene expression levels in response to some abiotic stresses and light treatment, obtained from the literature (*15*), are compared with the expression levels in the response to hypergravity ($N = 3$). The abiotic stresses were dehydration, heat stress and cold stress. The light treatments are 2 weeks of darkness, red light, blue light, UV-B or far-red light. Mean values of $\log_2$ (fold change) are shown for the eight AP2/ERF TFs that were upregulated by hypergravity in the present study, as well as the two AP2/ERF TFs that belong to the DREB or ERF subfamilies, respectively.

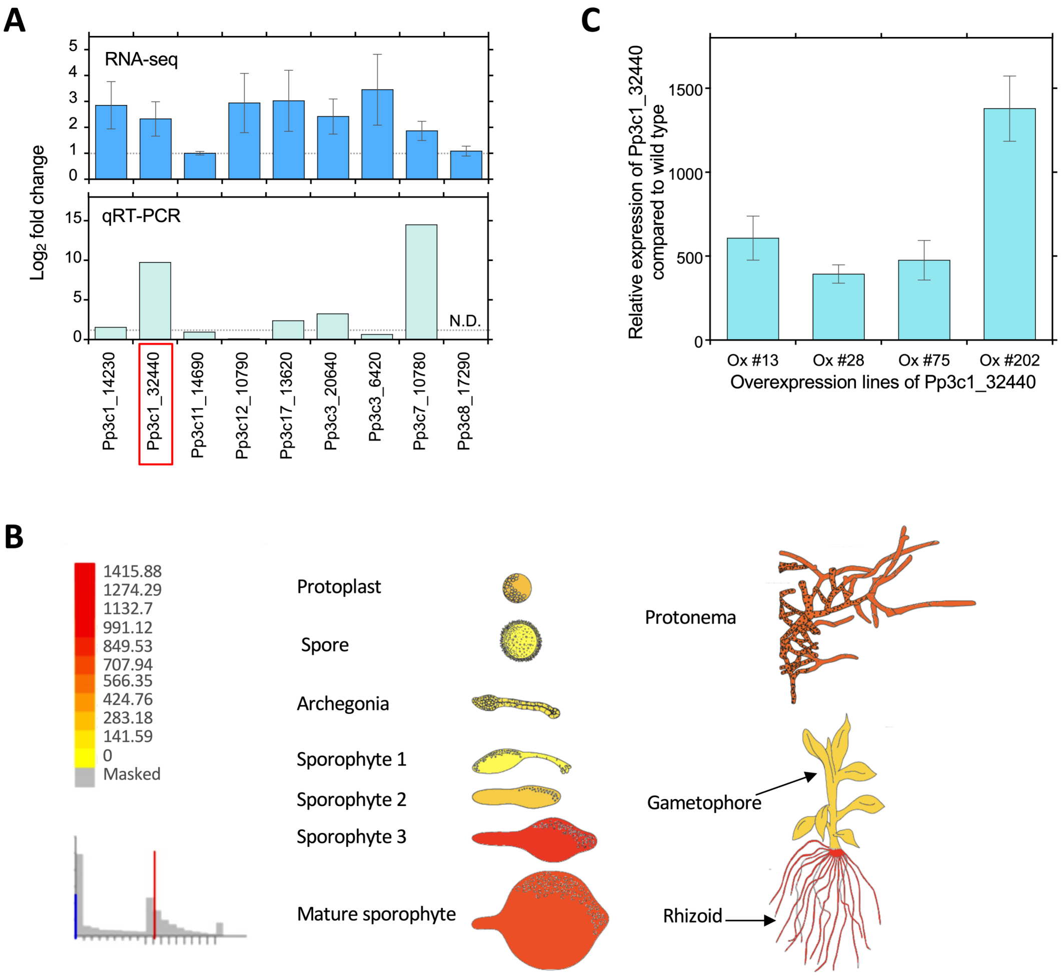

Fig. S3. Expression of *Pp3c1_32440* TF in wild-type *Physcomitrium patens* under hypergravity (10*g*) conditions, in *Pp3c1_32440* overexpression lines, and across various tissues. (A) Mean ± SE values ($N = 3$) of the $\log_2$ fold changes in the nine transcription factors (TFs) including the eight APETALA2/ETHYLENE-RESPONSIVE FACTOR (AP2/ERF) that were upregulated under 10*g* compared with 1*g*. The results of the qRT-PCR are compared with those from RNA-seq. *Pp3c8_17290* is not the AP2/ERF family, so no measurement by qRT-PCR was performed (N.D.). **(B)** Tissue-specific expression patterns of *Pp3c1_32440* TF. Predicted expression data were obtained from the *P. patens* eFP browser, illustrating distinct spatial expression across various developmental stages. **(C)** Expression of *Pp3c1_32440* in the four *Pp3c1_32440* overexpression lines (#13, #28, #75 and #202) generated in *Physcomitrium patens* under the control of the *PpEF1* promoter. The protonemata of the overexpression lines were cultured for 5 days on BCDAT agar medium. Transcript levels were normalized against the ubiquitin conjugating enzyme E2 gene *Pp3c14_21480v3.1*. The expression is presented relative to the levels in the wild type (untransformed plants). Data are presented as the means ± SE, based on three independent experiments ($N = 3$) of three biological replicates each.

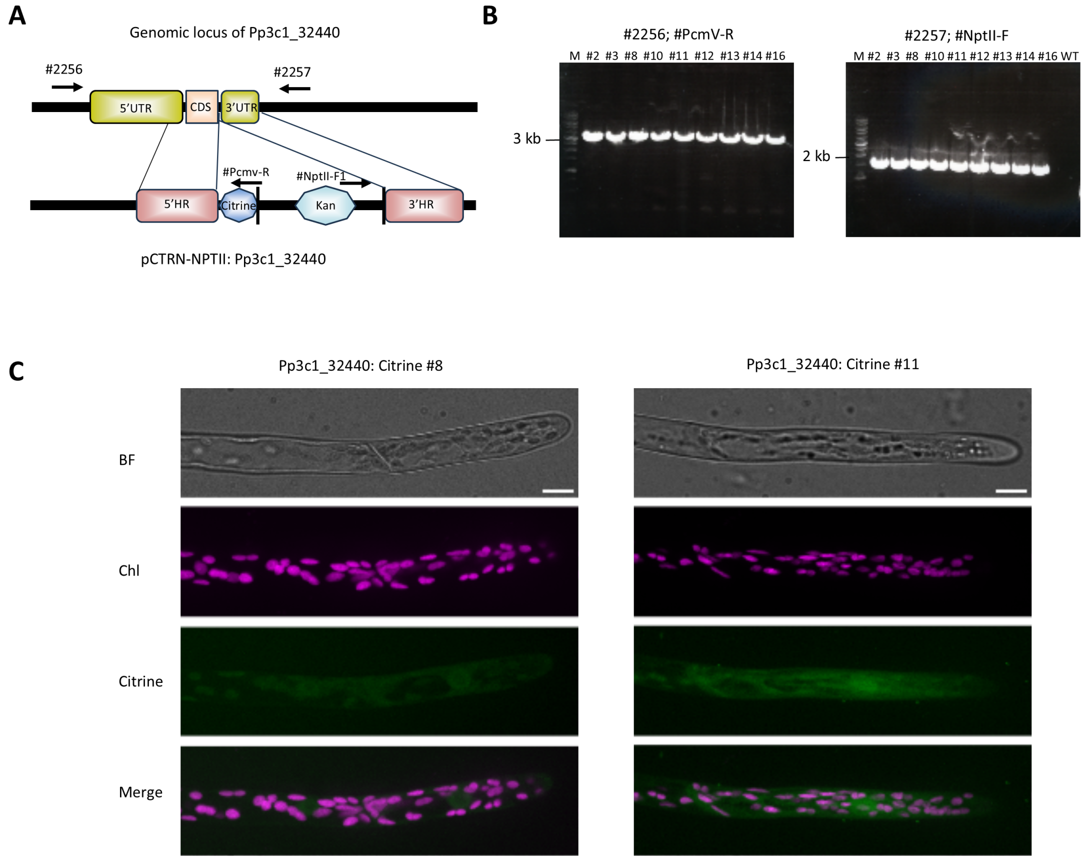

Fig. S4 Strategy, genotyping, and localization of Pp3c1_32440 in *Pp3c1_32440: Citrine* knock-in lines. (A) Schematic representation of the homologous recombination strategy used to generate the native promoter-driven *Pp3c1_32440: Citrine* knock-in reporter line. The approach involved cloning the 5' homologous region (HR), containing part of the *Pp3c1_32440* coding sequence (CDS), and the 3' HR, containing the stop codon and 3' UTR, into the pCTRN-NPTII vector to create pCTRN-NPTII-Pp3c1_32440. The pCTRN-NPTII plasmid confers G418 resistance in plants. Genotyping primers (indicated by arrows) were used to verify correct homologous recombination events. **(B)** PCR validation of independent transgenic lines to confirm successful recombination. The expected PCR product sizes were 3215 bp for primer pair #2256, Pcmv-R and 1709 bp for primer pair #2257, NptII-F1. Wild type (WT) served as a negative control. **(C)** Confocal images of 5-day-old protonemal cells from *Pp3c1_32440: Citrine* knock-in lines #8 and #11, showing the subcellular localization of the Pp3c1_32440: Citrine fusion protein. Citrine fluorescence indicates localization in both the cytosol and nucleus of protonemal cells. Scale bar = 20 μm.

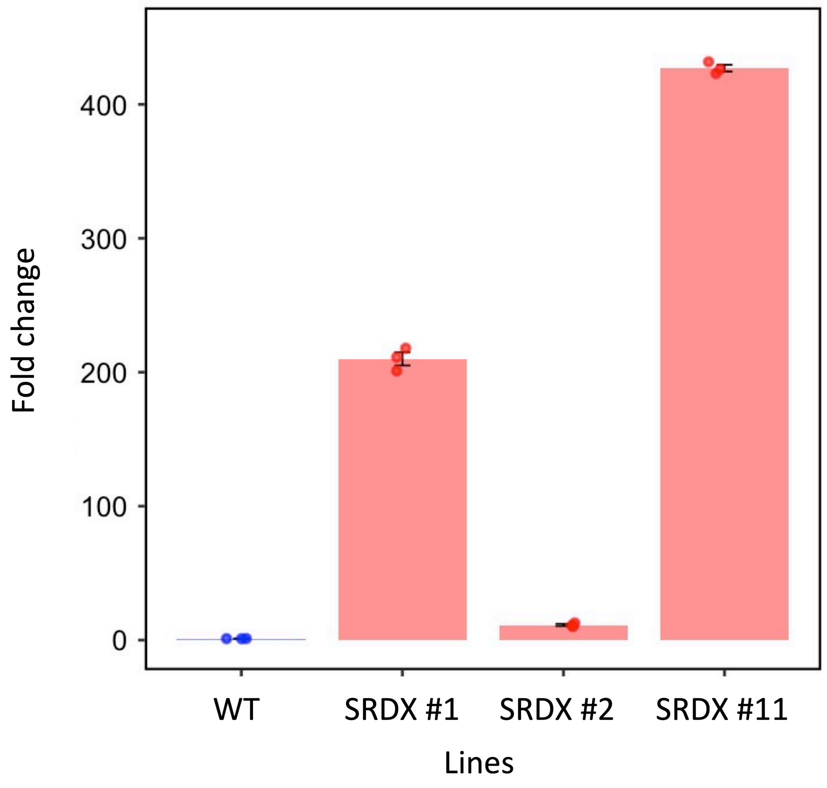

Fig. S5. Quantitative RT-PCR analysis in *Pp3c1_32440-SRDX* lines #1, #2 and #11.
Expression levels of *Pp3c1_32440* in wild-type (WT) and three independent transgenic lines were examined at 1 day after treatment with 1μM β-estradiol or DMSO. Expression levels were normalized to those of the DMSO-treated samples, which were set to 1. Ubiquitin-Conjugating Enzyme E2 was used as the reference gene for normalization. Data represent means ± SD from three biological replicates.

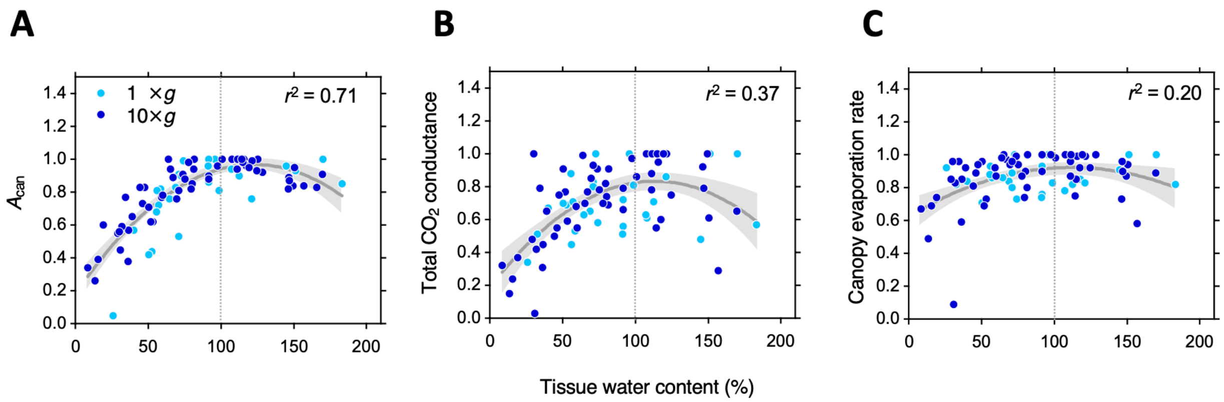

Fig. S6. Determination of photosynthesis parameters. Canopy photosynthesis rate (A_{can}) (**A**), canopy total CO_2 conductance (**B**) and canopy evaporation rate (**C**) against the tissue water content for the drying cycles (4–5 hours) of the *Physcomitrium patens* gametophores. The ratios of the parameters to their maximum values for each drying cycle were pooled for 1*g* (four cycles) and 10*g* (seven cycles). The data were fitted with a quadratic function (solid lines), with the r^2 values shown. Dashed lines indicate a 100% tissue water content. Measurements were performed under saturated irradiance (500 µmol m^{-2} s^{-1}) at a temperature of 25°C, a vapor pressure deficit of 1.0 kPa and an ambient CO_2 concentration of 400 ppm. Gray areas show 95% confidence intervals.

Table S1. Absolute values of the traits of *Physcomitrium patens* obtained from the hypergravity experiments.

	3*g* experiment		6*g* experiment		10*g* experiment	
Canopy and leaf traits	1*g*	3*g*	1*g*	6*g*	1*g*	10*g*
A_{can} (μmol m^{-2} s^{-1})	1.31 (0.08)	1.52 (0.07)$^{n.s.}$	0.82 (0.07)	1.24 (0.12)**	2.39 (0.12)<br>1.10 (0.17)	2.91 (0.02)***<br>1.72 (0.07)**
g_{can} (mmol m^{-2} s^{-1})	5.44 (0.48)	6.07 (0.22)$^{n.s.}$	2.47 (0.18)	3.84 (0.4)**	8.30 (0.27)<br>4.11 (0.65)	10.57 (0.22)***<br>6.02 (0.17)*
Canopy S_c (cm^2)	40.8 (0.4)	44.1 (1.2)$^{n.s.}$	31.4 (1.3)	68.5 (2.6)**	87.3 (1.4)<br>47.1 (1.8)	163.5 (5)**<br>80.9 (1.7)***
Canopy leaf area (cm^2)	46.6 (0.5)	50.8 (1.4)$^{n.s.}$	36.1 (1.5)	55.7 (2.1)**	89.7 (1.4)<br>48.4 (1.8)	123.3 (3.8)**<br>61.0 (1.3)**
Leaf S_c (m^2 m^{-2})	0.88 (0.17)	0.87 (0.09)$^{n.s.}$	0.87 (0.07)	1.23 (0.13)*	0.97 (0.09)	1.33 (0.11)*
Six potential key traits						
1. Cell wall thickness (μm)	0.404 (0.016)	0.393 (0.030)$^{n.s.}$	0.408 (0.032)	0.377 (0.018)$^{n.s.}$	0.338 (0.011)	0.321 (0.012)$^{n.s.}$
2. Chloroplast number (per cell)	18.8 (2.2)	19.8 (1.5)$^{n.s.}$	26.8 (2.5)	23.3 (0.2)$^{n.s.}$	18.5 (1.4)	22.0 (1.7)$^{n.s.}$
3. Chloroplast size (μm^2)	21.9 (0.9)	28.1 (1.2)***	22.7 (1.3)	33.5 (1.5)***	24.0 (1.2)	32.4 (1.6)***
4. Leaf area (mm^2)	0.818 (0.028)	0.864 (0.083)$^{n.s.}$	0.893 (0.043)	0.946 (0.031)$^{n.s.}$	0.638 (0.045)	0.677 (0.062)$^{n.s.}$
5. Leaf number (per gametophore)	32.5 (1.0)	31.5 (0.6)$^{n.s.}$	30.0 (1.5)	28.5 (1.2)$^{n.s.}$	28.0 (2.9)	26.5 (1.2)$^{n.s.}$
6. Gametophore number (per canopy)	175 (2)	187 (5)$^{n.s.}$	140 (6)	208 (8)**	502 (8)<br>271 (10)	708 (22)**<br>350 (8)**

Mean (SE) values are shown for the canopy gas exchange traits, such as canopy photosynthesis (A_{can}) and canopy CO_2 diffusional conductance (g_{can}; N = 10–18), canopy traits such as the surface area of chloroplasts (S_c) per canopy and the canopy leaf area (N = 3), and leaf traits such as leaf S_c (n = 6). Similarly, mean (SE) values are shown for the six potential key traits (Fig. 1B), such as cell wall thickness of the leaf cells (n = 40), chloroplast number in the leaf cells (N = 6), chloroplast size in the leaf cells (N = 98–161), leaf area (N = 12), leaf number (N = 4–12) and gametophore numbers per canopy (N = 3).

The 10*g* experiments were performed twice, with the mean values of the canopy traits obtained for each 10*g* experiment. A statistical analysis of the data from the 1*g* and hypergravity conditions was performed using Welch's *t* test, for which the significance of the differences is shown as * $P < 0.05$, ** $P < 0.01$ and *** $P < 0.001$. n.s. indicates no significant difference between conditions ($P > 0.05$).

Table S2. Gene Ontology (GO) enrichment analysis of the upregulated and downregulated genes in 10*g*-treated *Physcomitrium patens.*

GO term	Term type	Description	Enrichment FDR (adjusted *P*)	Number in input list	Number in the genome	Fold enrichment	Genes
**Upregulated**							
GO:0003700	F	DNA-binding transcription factor activity	0.002	9	640	5.8	Group1
GO:0006355	P	Regulation of transcription, DNA-templated	0.037	9	1183	3.1	Group1
GO:0072593	P	Reactive oxygen species metabolic process	0.037	3	102	12.2	PP3C12_6740, PP3C17_14500, PP3C17_14510
GO:0140110	F	Transcription regulator activity	0.005	9	772	4.8	Group1
GO:1903506	P	Regulation of nucleic acid–templated transcription	0.037	9	1183	3.1	Group1
GO:2001141	P	Regulation of RNA biosynthetic process	0.037	9	1183	3.1	Group1
**Downregulated**							
GO:0031967	L	Organelle envelope	0.017	4	853	9.4	Group2
GO:0031975	L	Envelope	0.017	4	853	9.4	Group2
GO:0042221	P	Response to chemical	0.049	3	577	10.5	PP3C13_5870, PP3C18_13590, PP3C9_1160

Group1: PP3C11_14690, PP3C12_10790, PP3C17_13620, PP3C1_14230, PP3C1_32440, PP3C2_11580, PP3C3_20640, PP3C3_6420 and PP3C7_10780; group2: PP3C10_5720, PP3C13_5870, PP3C24_720 and PP3C2_36220. The genes with a *P* value < 0.05 are shown. For the term types, P = biological process, F = molecular function

and L = cellular component. Number in input list means number of genes in DEGs belonging to the GO term, number in the genome means number of genes in the genome belonging to the GO term, and fold enrichment is defined as the percentage of genes in the DEGs belonging to a GO term, divided by the corresponding percentage in the genome.

Table S3. Differentially expressed genes between the 10*g*- and 1*g*-treated *Physcomitrium patens* plants.

Feature ID	RPKM 1*g* mean (SE)	RPKM 10*g* mean (SE)	$\log_2$ (fold change)	Encoded protein information based on Phytozome v13	Functions
Upregulated in 10*g*					
Pp3c1_10970	4.3(0.6)	11.6(4.0)	1.5*	Leucine-rich repeat N-terminal domain (LRRNT_2)	Others
**Pp3c1_14230**	**0.9(0.3)**	**6.2(2.0)**	**2.8*****	**AP2 domain (AP2)**	**TF**
Pp3c1_23040	0.5(0.1)	4.7(1.3)	3.3***	Protein tyrosine kinase (Pkinase_Tyr)/carbohydrate-binding protein of the ER (malectin_like)/leucine rich repeat (LRR_8)	PP
Pp3c1_30550	2.8(0.7)	15.6(6.1)	2.5***	Levansucrase/sucrose 6-fructosyltransferase	Others
**Pp3c1_32440**	**1.9(0.2)**	**9.6(4.5)**	**2.3*****	**AP2 domain (AP2)//C2H2-type zinc finger (zf-C2H2_6)**	**TF**
Pp3c1_34560	8.9(1.8)	19.3(3.1)	1.1**	Bestrophin, RFP-TM, chloride channel (Bestrophin)	TP
Pp3c1_35610	7.7(0.8)	20.4(3.8)	1.4***	Mitochondrial chaperone BCS1 (BCS1)	Others
Pp3c2_11580	3.5(0.4)	8.4(1.7)	1.3**	No data	*P. patens*-specific?
Pp3c2_24500	0.0(0.0)	1.2(0.8)	7.5***	Allene oxide cyclase (AOC)	SR
Pp3c2_29570	2.0(0.5)	10.6(6.0)	2.4*	Fructokinase (E2.7.1.4, scrK)	Others
Pp3c2_32640	4.8(0.2)	15.0(5.5)	1.6*	No data	*P. patens*-specific?
Pp3c3_1200	0.2(0.1)	2.3(1.1)	3.4**	No data	*P. patens*-specific?
Pp3c3_18090	0.2(0.0)	1.6(0.8)	2.9*	Glycogenin glucosyltransferase/priming glucosyltransferase, alpha-mannosyltransferase; predicted signal peptide and five predicted transmembrane helices	Others
**Pp3c3_20640**	**1.0(0.1)**	**5.2(2.4)**	**2.4****	**SHINE (SHN), DNA binding/transcription factor//subfamily not named**	**TF**
Pp3c3_21910	1.9(0.0)	13.1(6.4)	2.8***	Flotillin (FLOT)	Others
**Pp3c3_6420**	**0.2(0.0)**	**1.8(0.9)**	**3.5****	**AP2 domain (AP2)**	**TF**
Pp3c3_8160	0.4(0.1)	1.7(0.5)	2.1*	Predicted protein of unknown function; homologs in; identical to Phpat.003G028900 and Phpat.003G029200	Others
Pp3c4_10820	4.1(0.5)	10.2(3.1)	1.3*	Galactose oxidase/Beta-galactose oxidase	Others
Pp3c4_1261	2.7(0.3)	5.9(1.2)	1.1*	WD domain, G-beta repeat (WD40)//U-box domain (U-box)	Others
Pp3c4_3600	0.0(0.0)	1.7(1.0)	6.6**	Chitinase (E3.2.1.14)	Others
Pp3c5_17880	1.9(0.2)	5.1(1.1)	1.4**	Protein kinase domain (Pkinase)//legume lectin domain (Lectin_legB)	PP
Pp3c5_22500	3.3(0.6)	16.3(7.6)	2.3***	Predicted protein of unknown function; predicted transmembrane helix. *Physcomitrium* only	Others
Pp3c6_25120	0.3(0.0)	3.1(1.9)	3.2**	No data	*P. patens*-

					specific?
Pp3c6_5150	3.5(1.1)	11.8(2.4)	1.7**	Heavy metal transport/detoxification domain-containing protein	TP
**Pp3c7_10780**	**2.1(0.2)**	**7.5(2.1)**	1.9**	**AP2 domain (AP2)**	**TF**
Pp3c7_20720	0.5(0.2)	5.7(2.6)	3.4***	Cache domain (Cache_1)//von Willebrand factor type A domain (VWA_2), voltage-dependent calcium channel subunit alpha-2/delta-related	TP
**Pp3c8_17290**	**2.8(0.6)**	**6.0(0.8)**	**1.1***	**Transcription factor NAI1 (Basic helix-loop-helix protein 20)**	**TF**
Pp3c8_17610	0.6(0.1)	4.9(2.0)	3***	No data	*P. patens*-specific?
Pp3c8_19920	1.1(0.1)	8.1(3.5)	2.9***	Conserved plant protein of unknown function; predicted transmembrane helix	Others
Pp3c8_19930	0.8(0.3)	6.5(3.8)	3.1**	No data	*P. patens*-specific?
Pp3c8_20410	0.6(0.1)	5.6(3.3)	3.2**	Carboxymethylenebutenolidase homolog	Others
Pp3c8_2260	0.7(0.3)	10.5(5.2)	3.9***	Alpha/beta-hydrolases superfamily protein	Others
Pp3c8_4130	6.4(1.4)	21.9(5.7)	1.8***	Protein plant cadmium resistance 2-related	SR
Pp3c9_14890	0.9(0.3)	4.1(1.8)	2.2*	No data	ND
Pp3c9_17220	2.8(0.4)	11.2(4.0)	2.0***	Guard cell S-type anion channel SLAC1, enables the transmembrane transfer of an anion by a voltage-gated channel	TP
Pp3c10_1580	4.2(0.6)	8.8(1.5)	1.1**	F-box associated ubiquitination effector family protein-related	Others
Pp3c10_20350	32.1(5.9)	104.1(45.7)	1.7*	Ubiquitin//subfamily not named	Others
Pp3c10_21680	0.3(0.0)	1.2(0.3)	1.9**	Disease resistance protein-related	SR
Pp3c11_13030	1.5(0.4)	8.7(5.4)	2.5*	C2H2-type zinc finger (zf-C2H2_6)	Others
Pp3c11_14310	0.8(0.1)	5.7(2.8)	2.8***	No data	*P. patens*-specific?
Pp3c11_14600	0.2(0.1)	2.9(1.5)	4.0***	Actin cross-linking protein	TP
**Pp3c11_14690**	**5(0.1)**	**9.9(1.1)**	**1.0***	**AP2 domain (AP2)**	**TF**
Pp3c11_18000	9.2(0.9)	33.9(14.5)	1.9**	Nitrate, formate, iron dehydrogenase/subfamily not named	Others
Pp3c11_21880	0.3(0.0)	1.3(0.1)	2.3**	No data	*P. patens*-specific?
Pp3c11_26420	5.5(0.4)	13.2(3.7)	1.3*	No data	*P. patens*-specific?
Pp3c11_4360	0.9(0.1)	4.1(2.2)	2.3*	Subtilisin-like protease	Others
Pp3c11_5850	2.0(0.4)	8.3(3.7)	2.1**	Enhancer of mRNA-decapping protein 4 (EDC4)	Others
**Pp3c12_10790**	**0.2(0.0)**	**1.4(0.8)**	**2.9***	**AP2 domain (AP2)**	**TF**
Pp3c12_17290	0.0(0.0)	1.4(0.9)	7.7***	No data	*P. patens*-specific?
Pp3c12_2140	0.5(0.1)	1.9(0.4)	2.0**	Leucine-rich repeat protein kinase-like protein	PP
Pp3c12_6740	0.4(0.1)	4.2(2.8)	3.6**	Peroxidase (E1.11.1.7)	CW

Pp3c14_10610	0.0(0.0)	7.6(4.0)	10.1^{***}	INO80 complex subunit C (INO80C, IES6)	Others
Pp3c14_15500	0.0(0.0)	1.3(0.9)	5.8^{*}	No data	*P. patens*-specific?
Pp3c15_8300	0.0(0.0)	3.5(2.1)	9.0^{***}	No data	*P. patens*-specific?
Pp3c16_9400	10.3(0.5)	24.9(8)	1.3^{*}	Hypersensitive-induced response protein 1	Others
Pp3c17_13150	0.9(0.4)	3.1(0.4)	1.8^{*}	Coexpressed with genes in gametophores specific coexpression subnetwork	Others
**Pp3c17_13620**	**0.6(0.1)**	**4.5(2.9)**	**3.0^{*}**	**AP2 domain (AP2)//C2H2-type zinc finger (zf-C2H2_6)**	**TF**
Pp3c17_14500	4.1(1.5)	30.6(12.4)	2.9^{***}	Copper chaperone for superoxide dismutase (CCS)	SR
Pp3c17_14510	2.0(0.7)	16.4(7.0)	3.1^{***}	Superoxide dismutase [FE] 2, chloroplastic	CH
Pp3c17_14850	8.4(0.4)	19.3(3.0)	1.2^{***}	Nucleotide-diphospho-sugar transferase domain-containing protein-related	Others
Pp3c17_17450	0.9(0.1)	10.7(6.9)	3.6^{***}	Predicted protein of unknown function; large family in Physcomitrella only	Others
Pp3c17_18300	0.3(0.1)	1.8(0.7)	2.5^{*}	No data	*P. patens*-specific?
Pp3c17_5160	0.5(0.2)	3.3(1.6)	2.9^{*}	Pathogenesis-related thaumatin-like protein-related	SR
Pp3c18_18530	455.9(92.4)	3253.7(1568.8)	2.8^{***}	Predicted protein of unknown function; predicted signal peptide; family of three genes in Physcomitrella only	Others
Pp3c18_21090	0.9(0.5)	14.7(7.0)	4.0^{*}	Defense-related protein containing SCP domain, cysteine-rich secretory protein family	SR
Pp3c18_22120	3.6(0.8)	13.0(4.6)	1.8^{**}	Pectinesterase/pectinesterase inhibitor 16-related	CW
Pp3c19_10280	5.0(0.1)	11.1(3.1)	1.2^{*}	Transmembrane protein DDB_G0292058	Others
Pp3c19_14130	0.5(0.1)	2.2(0.7)	2.3^{**}	Alpha kinase/elongation factor 2 kinase//subfamily not named	PP
Pp3c19_6700	10.5(1.7)	75.9(43.6)	2.9^{***}	No data	*P. patens*-specific?
Pp3c20_20284	0.0(0.0)	2.7(0.8)	8.7^{***}	Rab3 GTPase-activating protein catalytic subunit (RAB3GAP1)	TP
Pp3c20_2930	1.0(0.3)	5.0(1.6)	2.4^{**}	Peptidase Do/Protease Do	Others
Pp3c23_12060	6.7(0.7)	16.1(4.7)	1.3^{*}	No data	*P. patens*-specific?
Pp3c23_15050	7.2(0.6)	17.4(5.6)	1.3^{*}	Rhomboid-related//subfamily not named	Others
Pp3c23_18360	0.8(0.1)	3.5(1.5)	2.2^{*}	Ubiquitin family (ubiquitin)	Others
Pp3c23_830	5.3(0.7)	13.2(2.1)	1.3^{***}	No data	*P. patens*-specific?
Pp3c24_5670	0.1(0.1)	4.4(1.6)	5.4^{***}	No data	*P. patens*-specific?
Pp3c26_1050	4.4(0.5)	9.6(2.0)	1.1^{**}	NIMA (never in mitosis)-related G2-specific serine/threonine protein kinase	PP

Pp3c26_8190	0.3(0.1)	2.6(1.3)	3.1**	Galactose oxidase/beta-galactose oxidase	Others
Pp3s123_10	72.7(8.5)	149.3(19.5)	1.0**	von Willebrand factor type A domain (VWA_2)	Others
Downregulated in 10*g*					
Pp3c2_16510	13.8(2.7)	5.3(1.6)	–1.4*	Coexpressed with genes in leaflets specific coexpression subnetwork	Others
Pp3c2_36220	121.1(16.3)	58.5(12.7)	–1.1*	Light-harvesting complex II chlorophyll a/b binding protein 2 (LHCB2)	CH
Pp3c3_36976	1.4(0.2)	0.5(0.0)	–1.6**	Pumilio RNA-binding family (PUM)	Others
Pp3c6_6030	1.6(0.8)	0.0(0.0)	–7.9**	Uncharacterized protein (K07053)	Others
Pp3c8_20950	2.2(0.7)	0.1(0.1)	–5.1***	LYR motif-containing protein 2	Others
Pp3c9_1160	3.1(0.3)	0.7(0.1)	–2.1***	Dual specificity protein phosphatase	PP
Pp3c10_5720	1.0(0.7)	0.0(0.0)	–7.3**	NADH dehydrogenase (ubiquinone) 1 alpha subcomplex subunit 1 (NDUFA1)	Others
Pp3c13_5870	26.1(4.3)	8.5(1.3)	–1.6***	Glutathione *S*-transferase-related	Others
Pp3c16_9850	2.0(1.1)	0.0(0.0)	–8.3***	No data	*P. patens*-specific?
Pp3c17_931	2.9(0.7)	0.0(0.0)	–8.7***	BTB/POZ and math domain-containing protein 1-related	Others
Pp3c18_13590	11.5(2.3)	5.4(0.3)	–1.1**	Catalase (katE, CAT, catB, srpA)	Others
Pp3c20_7590	34.6(12.0)	9.0(1.0)	–1.9***	*S*-phase kinase-associated protein 1 (SKP1, CBF3D)	PP
Pp3c22_12780	32.7(3.1)	15.4(2.2)	–1.1***	*S*-adenosylmethionine synthetase (metK)	Others
Pp3c24_720	57.6(13.7)	28.1(2.7)	–1.0*	CDGSH-type Zn-finger containing protein	Others
Pp3c26_4630	11.5(2.3)	2.5(0.4)	–2.2***	Phosphoenolpyruvate carboxykinase (GTP)/phosphopyruvate carboxylase	CH
Pp3c27_7650	16.7(1.8)	7.0(1.1)	–1.3**	Predicted membrane protein (DUF2306)	Others

Boldface symbols indicate genes encoding transcription factors. The mean (SE) reads per kilobase per million reads (RPKM) values and $\log_2$(fold change) ($N = 3$) are shown. The data were selected such that either averaged 1*g* data or averaged 10*g* data had an RPKM > 1, with the results of the EDGE test (BH method) between 1*g* and 10*g* being $^{*}P < 0.05$, $^{**}P < 0.01$ and $^{***}P < 0.001$. For the gene functions, TF = transcription factors, PP = protein phosphorylation, SR = stress response, CW = cell wall, CH = related to chloroplast and TP = related to transport. The database used was Phytozome 13.

Table S4. Top hit lists of *Arabidopsis thaliana* AP2/ERF transcription factors searched by the BLASTP program using the AP2 domains of the eight AP2/ERF transcription factors in *Physcomitrium patens* as queries.

Query	Best hit AGI code	Subfamily	Identity/similarity (%)
Pp3c1_14230	AT1G03800	ERF	52/76
Pp3c1_32440	AT2G36450	DREB	56/68
Pp3c3_20640	AT2G35700	DREB	55/63
Pp3c3_6420	AT5G21960	DREB	51/65
Pp3c7_10780	AT1G19210	DREB	56/77
Pp3c11_14690	AT1G19210	DREB	56/77
Pp3c12_10790	AT4G31060	DREB	51/68
Pp3c17_13620	AT2G36450	DREB	56/68

Table S5. Absolute values of the traits of *Physcomitrium patens* obtained from the experiments of *Pp3c1_32440*-overexpressing lines.

	Experiment 1			Experiment 2		
Canopy and leaf traits	Control	Ox#13	Ox#202	Control	Ox#28	Ox#75
A_{can} (µmol m^{-2} s^{-1})	1.69 (0.12)	2.13 (0.08)***	2.05 (0.07) **	2.19 (0.08)	3.99 (0.33) ***	3.56 (0.1) ***
g_{can} (mmol m^{-2} s^{-1})	5.1 (0.3)	6.0 (0.1) **	5.4 (0.2) n.s.	7.4 (0.3)	15.5 (1.3) ***	12.2 (0.2) ***
Canopy S_c (cm^2)	32 (2)	64 (6) **	48 (7) *	112 (5)	160 (6) ***	192 (7) ***
Canopy leaf area (cm^2)	171 (14)	250 (25) n.s.	223 (31) n.s.	610 (28)	658 (28) **	717 (24) ***
Leaf S_c (m^2 m^{-2})	0.82 (0.07)	1.59 (0.06) ***	1.47 (0.16) **	1.07 (0.08)	1.46 (0.06)**	1.92 (0.08) ***
Six potential key traits						
1. Cell wall thickness (µm)	0.48 (0.02)	0.48 (0.03) n.s.	0.37 (0.01) n.s.	0.37 (0.01)	0.38 (0.02) n.s.	0.44 (0.02) n.s.
2. Chloroplast number (per cell)	22.5 (1.8)	19.6 (1.2) n.s.	20.3 (0.7) n.s.	32.5 (1.3)	29.9 (1.5) n.s.	31.6 (1.4) n.s.
3. Chloroplast size (µm^2)	10.2 (0.2)	16.4 (0.4) ***	18.9 (0.5) ***	9.9 (0.2)	12.9 (0.3) ***	15.9 (0.3) ***
4. Leaf area (mm^2)	0.559 (0.024)	0.558 (0.021) n.s.	0.490 (0.025) n.s.	0.814 (0.037)	0.662 (0.019)***	0.777 (0.022) n.s.
5. Leaf number (per gametophore)	24.8 (0.8)	22.9 (0.8) n.s.	21.3 (0.8) *	24.8 (0.7)	22.3 (0.8) n.s.	23.4 (0.9) n.s.
6. Gametophore number (per canopy)	227 (14)	363 (35) *	293 (40) n.s.	484 (22)	699 (29) ***	631 (21) ***

Mean (SE) values are shown for the canopy gas exchange traits, such as canopy photosynthesis (A_{can}) and canopy CO_2 diffusional conductance (g_{can}; N = 12–18), canopy traits such as the surface area of chloroplasts (S_c) per canopy and canopy leaf area (n = 6–12), and leaf traits such as leaf S_c (N = 6). Similarly, mean (SE) values are shown for the six potential key traits (Fig. 1b) such as cell wall thickness of the leaf cells (N = 40), chloroplast number in the leaf cells (N = 15), chloroplast size in the leaf cells (N = 217–380), leaf area (N = 30), leaf numbers (N = 10–15) and gametophore numbers per canopy (N = 6–12). A statistical analysis was performed between the control and overexpression plants using Welch's t test, for which the significance of the differences is shown as * $P < 0.05$, ** $P < 0.01$ and *** $P < 0.001$. n.s. indicates no significant difference between conditions ($P > 0.05$).

Table S6 (A). Shoot length of the gametophores of *Physcomitrium patens* obtained from the experiments of hypergravity, *Pp3c1_32440*-overexpressing lines, and *Pp3c1_32440-SRDX* lines.

Experiment	Ratio	Absolute values (mm)
Hypergravity		
3*g*	0.78(0.02)***	1*g*: 1.60(0.03) 3*g*: 1.25(0.03)
6*g*	0.82(0.05)***	1*g*: 1.06(0.05) 6*g*: 0.87(0.05)
10*g*	0.84(0.03)***	1*g*: 0.70(0.03) 10*g*: 0.58(0.02)
Overexpressing Pp3c1_32440		
Wild type	-	4.53(0.12)
#13	0.61(0.02)***	2.83(0.10)
#28	0.64(0.03)***	2.84(0.11)
#75	0.66(0.04)***	2.92(0.16)
#202	0.54(0.02)***	2.49(0.10)
Pp3c1_32440-SRDX		
1*g*		
Wild type	-	3.47(0.09)
#1	0.96(0.03)n.s.	DMSO: 3.25(0.09) EST: 3.12(0.09)
#2	0.97(0.02)n.s.	DMSO: 3.10(0.09) EST: 2.99(0.07)
#11	1.09(0.03)**	DMSO: 3.24(0.06) EST: 3.53(0.09)
10*g*		
Wild type	-	1.15(0.04)
#1	1.43(0.05)***	DMSO: 1.19(0.06) EST: 1.70(0.06)
#2	1.43(0.09)***	DMSO: 1.31(0.07) EST: 1.88(0.12)
#11	1.33(0.05)***	DMSO: 1.18(0.05) EST: 1.58(0.06)

The ratios were calculated such that 1) to the plants grown under 1*g* for the experiments of hypergravity, 2) to the wild type plants for the experiments of *Pp3c1_32440*-overexpressing lines, and 3) to DMSO treatment plants for the experiments of *Pp3c1_32440-SRDX* lines. Mean (SE) values of the ratios and absolute values are shown ($N = 20$–31). A statistical analysis was performed between the 1*g* and hypergravity-grown plants, between the wild type and *Pp3c1_32440*-overexpressing lines and between DMSO-treated and EST-treated plants, respectively, using Welch's *t* test, for which the significance of the differences is shown as ** $P < 0.01$, *** $P < 0.001$ and n.s. not significant ($P > 0.05$).

Table S6 (B). Absolute values of the traits of *Physcomitrium patens* obtained from the experiments of *Pp3c1_32440-SRDX* lines.

Traits / Lines		1*g* DMSO	1*g* EST	10*g* DMSO	10*g* EST	Two-way ANOVA Treatment	Two-way ANOVA Gravity	Two-way ANOVA Treatment × gravity
$A_{chloronema}$ (μm m^{-2} s^{-1})								
	Wild type	0.722 (0.009)	0.780 (0.012)	0.770 (0.047)	0.747 (0.018)	n.s.	n.s.	n.s.
	SRDX					**	***	*
	#1	0.915 (0.066)	0.466 (0.022)	0.684 (0.030)	0.532 (0.036)			
	#2	0.600 (0.01)	0.584 (0.023)	0.470 (0.010)	0.567 (0.034)			
	#11	0.628 (0.031)	0.558 (0.035)	0.621 (0.031)	0.474 (0.013)			
Chloroplast size ($μm^2$)								
	Wild type	19.5 (1.2)	20.7 (1.0)	24.5 (1.3)	25.6 (1.0)	n.s.	***	n.s.
	SRDX					***	***	*
	#1	26.2 (1.6)	11.2 (0.8)	30.9 (2.1)	16.5 (0.9)			
	#2	24.4 (1.4)	18.1 (0.9)	19.0 (1.2)	25.2 (1.6)			
	#11	21.8 (1.1)	10.6 (0.6)	25.5 (1.5)	13.3 (0.7)			
Plant dry mass (mg)								
	Wild type	31.9 (1.8)	29.0 (0.9)	21.1 (1.8)	24.6 (4.0)	n.s.	*	n.s.
	SRDX					n.s.	n.s.	n.s.
	#1	26.7 (3.1)	17.5 (2.5)	18.6 (3.0)	18.3 (2.8)			
	#2	19.7 (0.7)	27.8 (3.5)	19.2 (2.5)	16.3 (4.2)			
	#11	25.8 (2.1)	21.3 (0.7)	24.7 (4.2)	25.9 (1.6)			

Mean (SE) values are shown for the canopy gas exchange traits, such as chloronema photosynthesis ($A_{chloronema}$)($N = 9$), chloroplast size ($N = 33$–49), and plant dry mass ($N = 3$). The effect of gravity and treatment was tested using two-way ANOVA for wild type and three *Pp3c1_32440-SRDX* lines, respectively, for which the significant levels of the effects are shown as * $P < 0.05$, ** $P < 0.01$, *** $P < 0.001$ and n.s.; not significant ($P > 0.1$).

Table S7. Photosynthesis and plant traits in comparison with previous studies of bryophytes.

	Present study		Previous studies
Traits	*Physcomitrium patens* (1*g*) mean (SE)	*N*	Bryophytes min–max
Canopy photosynthesis traits			
A_{can} (μmol m^{-2} s^{-1})	1.57(0.10)	62	0.37 – 2.03 (*56*), 0.59 – 1.41 (*14*), 0.43 – 5.71 (*7*), 0.44 – 4.22 (*50*)
g_{can} (mmol m^{-2} s^{-1})	5.70(0.34)	62	1.86 – 22.5 (*7*), 3 – 14 (*57*), 10 – 70# (*50*)
Leaf traits			
Leaf area (mm^2)	0.783(0.029)	36	0.28 – 7.71 (*14*)
Lamina cell numbers	12.5(0.8)	20	10.0 – 14.4 (*8*)
Lamina thickness (μm)	24.7(1.2)	48	23.6 – 26.1 (*8*), 6.7 – 47.5 (*14*)
Lamina cell diameter (μm)	23.0(1.2)	24	21.2 –32.0 (*8*), 6 – 195 (*14*)
Leaf S_c (m^2 m^{-2})	0.907(0.066)	18	0.59 – 5.18 (*7*)
Cell wall thickness (μm)	0.384(0.013)	120	0.167 – 0.386 (*8*), 0.97 – 5.04 (*14*), 0.50 – 3.41 (*7*)
Chloroplast diameter (μm)	3.78(0.08)	377	0.80 – 10.97 (*8*), 2.05 – 7.28 (*7*)
Chloroplast thickness (μm)	1.82(0.03)	377	0.76 – 4.03 (*8*), 0.89 – 4.17 (*7*)

Canopy photosynthesis traits and leaf traits for the present study were pooled for the 1*g* controls. Canopy traits include canopy photosynthesis (A_{can}) and canopy CO_2 diffusional conductance (g_{can}). Leaf traits, including the surface area of chloroplasts (S_c) per leaf, are also shown. Leaf area was analyzed using fresh gametophores, while the other anatomical traits were analyzed using light micrographs of sections embedded in Spurr's resin. Data from previous studies are also shown. # Data obtained using the chlorophyll fluorescence method.

Table S8. Primers used in this study.

No	Primer name	Sequence (5'-3')
#234	PpE2-F1	TACGGACCCTAATCCAGATGAC
#235	PpE2-R1	CAACCCATTGCATACTTCTGAG
#275	Alpha-tubulin-R	GTACTCGTCGTCGTCCTGTCC
#308	Alpha-tubulin-seq-F	CACTGTTGTTGGAGAGGTTG
#573	Pp3c1_14230_f1	TTGAGCCCTCACATTCCAAGCC
#574	Pp3c1_14230_r1	TGAGCTTCGCTGGGTTGAGTTAG
#575	Pp3c3_6420_f1	GTCTCACGCAGCAATCTGAAGC
#576	Pp3c3_6420_r1	GTCGACTTTCATGAGCTGCCTAAC
#577	Pp3c3_20640_f1	AATGGCCGTAGTCCTACGAAACGC
#578	Pp3c3_20640_r1	TTCTGCAGATCTTGCCGGAATCG
#579	Pp3c1_32440_f1	TGCGAGGAGGACGTCAACAATTAC
#580	Pp3c1_32440_r1	TCAATATTTACCCGTGGCCAGAGC
#581	Pp3c12_10790_f1	AGATGCTCGGCGATGTTTCAGC
#582	Pp3c12_10790_r1	ACAATCCAAAGCACCACGACAC
#583	Pp3c7_10780_f1	AAACCAAGACCCACAGGACTCAC
#584	Pp3c7_10780_r1	GGTTTGCTACGCAACACAAGGG
#585	Pp3c17_13620_f1	TGGCGATGCAGTCAGATGGAAG
#586	Pp3c17_13620_r1	AGGATTGTACGCATTGCCAAACG
#621	NptII-F1	GCCCCCGCTTAAAAATTGGTATCAGAGCC
#901	Pp3c1_32440_entry_f1	AACCAATTCAGTCGACATGGCTTCTTCTGGCAGCGACA
#902	Pp3c1_32440_entry_r2	AAGCTGGGTCTAGATATCC CCCGTGGCCAGAGCCGTACTGCGC
#1215	Pp3c1_32440-Citrin-5'_ki_f1	CCCCCTCGAGGTCGACACACCTGCAATTGGCTGTAGACCTA
#1216	Pp3c1_32440-Citrin-5'_ki_r1	CTTGCTCACCAAGATATC CCTGTACATTTTGGACACGAGGA
#1217	Pp3c1_32440-Citrin-3'_ki_f1	GGGATCCACGTGCCCGGGTAAATATTGAATCTTAATCCACCTG
#1218	Pp3c1_32440-Citrin-3'_ki_r1	TGGCGGCCGCTCTAGACAAATTTTAAGGACTAATGAAC
#1372	Pcmv-R	GAGGAAGGGTCTTGCGAAGGATAGTG
#2256	Geno-Pp3c1_32440-F	CATCTGGGTGACACCTCATCATGAT
#2257	Geno-Pp3c1_32440-R	GGCATAAATTAACGATGTGGTGTTCATA
#2287	Pp3c1_32440-qPCR-F	TGCGAGGAGGACGTCAACAATTAC
#2288	Pp3c1_32440-qPCR-R	TCAATATTTACCCGTGGCCAGAGC
#2321	Pp3c1_32440-semiqPCR-F	ATGGCTTCTTCTGGCAGCGACAG
#2322	Pp3c1_32440-semiqPCR-R	CCCGTGGCCAGAGCCGTACTGCGC

All primers were used in this study for cloning, RT-PCR, RT-qPCR.

Other Supplementary Material for this manuscript includes the following:

1) **Data S1. Data for Fig 2 and table S1.** Absolute values of the traits of Physcomitrium patens obtained from the hypergravity experiments.

2) **Data S2. Data for Fig 3B and C.** (B), Results of the Gene Ontology (GO) enrichment analysis. The DEGs upregulated at 10g were annotated as six GO terms, five of which were related to transcription factors (TFs). (C), Reads per kilobase per million reads (RPKM) values at 1g and 10g are shown for the nine genes, including eight encoding the AP2/ERF transcription factors.

3) **Data S3. Data for Fig 4A.** Fold change (10g vs. 1g) of Pp3c1_32440 TF in protonemal cells and gametophores measured by quantitative RT-PCR analysis at three distinct time points.

4) **Data S4. Data for Fig. 5B and E and table S6(B)**. Absolute values of the photosynthetic, morphological and anatomical traits of the Pp3c1_32440-overexpressing lines and Pp3c1_32440-SRDX lines.

5) **Data S5. Data for Fig S2C.** The gene expression levels in response to some abiotic stresses and light treatment, obtained from the literature (15), are compared with the expression levels in the response to hypergravity.

6) **Data S6. Data for Fig S6.** Canopy photosynthesis rate (Acan), canopy total CO2 conductance (gt) and canopy evaporation rate (E) against the tissue water content for the drying cycles (4–5 hours) of the Physcomitrium patens gametophores.

7) **Data S7. Data for Table S6A.** Shoot length of the gametophores of Physcomitrium patens obtained from the experiments of hypergravity, Pp3c1_32440-overexpressing lines, and Pp3c1_32440-SRDX lines.

REFERENCES AND NOTES

1. T. Servais, B. Cascales-Miñana, C. J. Cleal, P. Gerrienne, D. A. T. Harper, M. Neumann, Revisiting the great Ordovician diversification of land plants: Recent data and perspectives. *Palaeogeogr. Palaeoclimatol. Palaeoecol.* **534**, 109280 (2019).

2. N. J. Wickett, S. Mirarab, N. Nguyen, T. Warnow, E. Carpenter, N. Matasci, S. Ayyampalayam, M. S. Barker, J. G. Burleigh, M. A. Gitzendanner, B. R. Ruhfel, E. Wafula, J. P. Der, S. W. Graham, S. Mathews, M. Melkonian, D. E. Soltis, P. S. Soltis, N. W. Miles, C. J. Rothfels, L. Pokorny, A. J. Shaw, L. DeGironimo, D. W. Stevenson, B. Surek, J. C. Villarreal, B. Roure, H. Philippe, C. W. DePamphilis, T. Chen, M. K. Deyholos, R. S. Baucom, T. M. Kutchan, M. M. Augustin, J. Wang, Y. Zhang, Z. Tian, Z. Yan, X. Wu, X. Sun, G. K.-S. Wong, J. Leebens-Mack, Phylotranscriptomic analysis of the origin and early diversification of land plants. *Proc. Natl. Acad. Sci. U.S.A.* **111**, E4859–E4868 (2014).

3. R. P. Hangarter, Gravity, light and plant form. *Plant Cell Environ.* **20**, 796–800 (1997).

4. P. Sarkar, E. Bosneaga, M. Auer, Plant cell walls throughout evolution: Towards a molecular understanding of their design principles. *J. Exp. Bot.* **60**, 3615–3635 (2009).

5. I. Terashima, Y. T. Hanba, D. Tholen, Ü. Niinemets, Leaf functional anatomy in relation to photosynthesis. *Plant Physiol.* **155**, 108–116 (2011).

6. T. Tosens, K. Nishida, J. Gago, R. E. Coopman, M. Cabrera, M. Carriquí, L. Laanisto, L. Morales, M. Nadal, R. Rojas, E. Talts, M. Tomas, Y. Hanba, Ü. Niinemets, J. Flexas, The photosynthetic capacity in 35 ferns and fern allies: Mesophyll CO_2 diffusion as a key trait. *New Phytol.* **209**, 1576–1590 (2016).

7. M. Carriquí, M. Roig-Oliver, T. J. Brodribb, R. Coopman, W. Gill, K. Mark, Ü. Niinemets, A. V. Perera-Castro, M. Ribas-Carbó, L. Sack, T. Tosens, M. Waite, J. Flexas, Anatomical constraints to nonstomatal diffusion conductance and photosynthesis in lycophytes and bryophytes. *New Phytol.* **222**, 1256–1270 (2019).

8. K. Takemura, H. Kamachi, A. Kume, T. Fujita, I. Karahara, Y. T. Hanba, Hypergravity environment increases chloroplast sizes, photosynthesis and plant growth of the moss *Physcomitrella patens*. *J. Plant Res.* **130**, 181–192 (2017).

9. D. M. Obenland, C. S. Brown, The influence of altered gravity on carbohydrate metabolism in excised wheat leaves. *J. Plant Physiol.* **144**, 696–699 (1994).

10. P. B. Vidyasagar, S. S. Jagtap, J. P. Dixit, S. M. Kamble, A. P. Dhepe, Effects of short-term hypergravity exposure on germination, growth and photosynthesis of *Triticum aestivum* L. *Microgravity Sci. Technol.* **26**, 375–384 (2014).

11. L. A. Moody, Unravelling 3D growth in the moss *Physcomitrium patens*. *Essays Biochem.* **66**, 769–779 (2022).

12. Y. Hata, J. Kyozuka, Fundamental mechanisms of the stem cell regulation in land plants: Lesson from shoot apical cells in bryophytes. *Plant Mol. Biol.* **107**, 213–225 (2021).

13. D. Tamaoki, I. Karahara, T. Nishiuchi, T. Wakasugi, K. Yamada, S. Kamisaka, Involvement of auxin dynamics in hypergravity-induced promotion of lignin-related gene expression in *Arabidopsis* inflorescence stems. *J. Exp. Bot.* **62**, 5463–5469 (2011).

14. M. Waite, L. Sack, How does moss photosynthesis relate to leaf and canopy structure? Trait relationships for 10 Hawaiian species of contrasting light habitats. *New Phytol.* **185**, 156–172 (2010).

15. P. Perroud, F. B. Haas, M. Hiss, K. K. Ullrich, A. Alboresi, M. Amirebrahimi, K. Barry, R. Bassi, S. Bonhomme, H. Chen, J. C. Coates, T. Fujita, A. Guyon-Debast, D. Lang, J. Lin, A. Lipzen, F. Nogué, M. J. Oliver, I. Ponce de León, R. S. Quatrano, C. Rameau, B. Reiss, R. Reski, M. Ricca, Y. Saidi, N. Sun, P. Szövényi, A. Sreedasyam, J. Grimwood, G. Stacey, J. Schmutz, S. A. Rensing, The *Physcomitrella patens* gene atlas project: Large-scale RNA-seq based expression data. *Plant J.* **95**, 168–182 (2018).

16. Y. Mizokami, R. Oguchi, D. Sugiura, W. Yamori, K. Noguchi, I. Terashima, Cost-benefit analysis of mesophyll conductance: Diversities of anatomical, biochemical and environmental determinants. *Ann. Bot.* **130**, 265–283 (2022).

17. S. Dutta, J. A. Cruz, S. M. Imran, J. Chen, D. M. Kramer, K. W. Osteryoung, Variations in chloroplast movement and chlorophyll fluorescence among chloroplast division mutants under light stress. *J. Exp. Bot.* **68**, 3541–3555 (2017).

18. B. Grigorova, V. Vassileva, D. Klimchuk, I. Vaseva, K. Demirevska, U. Feller, Drought, high temperature, and their combination affect ultrastructure of chloroplasts and mitochondria in wheat (*Triticum aestivum* L.) leaves. *J. Plant Interact.* **7**, 204–213 (2012).

19. Yu. V. Venzhik, A. F. Titov, V. V. Talanova, E. A. Miroslavov, Ultrastructure and functional activity of chloroplasts in wheat leaves under root chilling. *Acta Physiol. Plant.* **36**, 323–330 (2014).

20. B. Jin, L. Wang, J. Wang, K.-Z. Jiang, Y. Wang, X.-X. Jiang, C.-Y. Ni, Y.-L. Wang, N.-J. Teng, The effect of experimental warming on leaf functional traits, leaf structure and leaf biochemistry in *Arabidopsis thaliana*. *BMC Plant Biol.* **11**, 35 (2011).

21. R. Oguchi, K. Hikosaka, T. Hirose, Leaf anatomy as a constraint for photosynthetic acclimation: Differential responses in leaf anatomy to increasing growth irradiance among three deciduous trees. *Plant Cell Environ.* **28**, 916–927 (2005).

22. T. H. Do, P. Pongthai, M. Ariyarathne, O. K. Teh, T. Fujita, AP2/ERF transcription factors regulate salt-induced chloroplast division in the moss *Physcomitrella patens*. *J. Plant Res.* **133**, 537–548 (2020).

23. M. Carriquí, H. M. Cabrera, M. Conesa, R. E. Coopman, C. Douthe, J. Gago, A. Gallé, J. Galmés, M. Ribas-Carbo, M. Tomás, J. Flexas, Diffusional limitations explain the lower photosynthetic capacity of ferns as compared with angiosperms in a common garden study. *Plant Cell Environ.* **38**, 448–460 (2015).

24. K. W. Osteryoung, K. A. Pyke, Division and dynamic morphology of plastids. *Annu. Rev. Plant Biol.* **65**, 443–472 (2014).

25. J. de Keijzer, A. Freire Rios, V. Willemsen, *Physcomitrium patens*: A single model to study oriented cell divisions in 1D to 3D patterning. *Int. J. Mol. Sci.* **22**, 2626 (2021).

26. L. A. Moody, S. Kelly, E. Rabbinowitsch, J. A. Langdale, Genetic regulation of the 2D to 3D growth transition in the moss *Physcomitrella patens*. *Curr. Biol.* **28**, 473–478.e5 (2018).

27. T. Aoyama, Y. Hiwatashi, M. Shigyo, R. Kofuji, M. Kubo, M. Ito, M. Hasebe, AP2-type transcription factors determine stem cell identity in the moss *Physcomitrella patens*. *Development* **139**, 3120–3129 (2012).

28. S. Wang, Y. Guan, Q. Wang, J. Zhao, G. Sun, X. Hu, M. P. Running, H. Sun, J. Huang, A mycorrhizae-like gene regulates stem cell and gametophore development in mosses. *Nat. Commun.* **11**, 2030 (2020).

29. C. Hirt, S. Claessens, T. Fecher, M. Kuhn, R. Pail, M. Rexer, New ultrahigh-resolution picture of Earth's gravity field. *Geophys. Res. Lett.* **40**, 4279–4283 (2013).

30. K. A. Franklin, Shade avoidance. *New Phytol.* **179**, 930–944 (2008).

31. E. Afshinnekoo, R. T. Scott, M. J. MacKay, E. Pariset, E. Cekanaviciute, R. Barker, S. Gilroy, D. Hassane, S. M. Smith, S. R. Zwart, M. Nelman-Gonzalez, B. E. Crucian, S. A. Ponomarev, O. I. Orlov, D. Shiba, M. Muratani, M. Yamamoto, S. E. Richards, P. A. Vaishampayan, C. Meydan, J. Foox, J. Myrrhe, E. Istasse, N. Singh, K. Venkateswaran, J. A. Keune, H. E. Ray, M. Basner, J. Miller, M. H. Vitaterna, D. M. Taylor, D. Wallace, K. Rubins, S. M. Bailey, P. Grabham, S. V. Costes, C. E. Mason, A. Beheshti, Fundamental biological features of spaceflight: Advancing the field to enable deep-space exploration. *Cell* **183**, 1162–1184 (2020).

32. J. C. Mortimer, M. Gilliham, SpaceHort: Redesigning plants to support space exploration and on-earth sustainability. *Curr. Opin. Biotechnol.* **73**, 246–252 (2022).

33. V. Maiwald, K. Kyunghwan, V. Vrakking, C. Zeidler, From Antarctic prototype to ground test demonstrator for a lunar greenhouse. *Acta Astronaut.* **212**, 246–260 (2023).

34. S. A. Narayanan, Gravity's effect on biology. *Front. Physiol.* **14**, 1199175 (2023).

35. A. Kume, H. Kamachi, Y. Onoda, Y. T. Hanba, Y. Hiwatashi, I. Karahara, T. Fujita, How plants grow under gravity conditions besides 1 g: Perspectives from hypergravity and space experiments that employ bryophytes as a model organism. *Plant Mol. Biol.* **107**, 279–291 (2021).

36. D. Qiu, Y. Jian, Y. Zhang, G. Xie, Plant gravitropism and signal conversion under a stress environment of altered gravity. *Int. J. Mol. Sci.* **22**, 11723 (2021).

37. T. Nishiyama, Y. Hiwatashi, I. Sakakibara, M. Kato, M. Hasebe, Tagged mutagenesis and gene-trap in the moss, *Physcomitrella patens* by shuttle mutagenesis. *DNA Res.* **7**, 9–17 (2000).

38. A. Mori, H. Kamachi, I. Karahara, A. Kume, Y. T. Hanba, K. Takemura, T. Fujita, Comparisons of the effects of vibration of two centrifugal systems on the growth and morphological parameters of the moss *Physcomitrella patens*. *Biol. Sci. Space* **31**, 9–13 (2017).

39. M. Kawase, Y. T. Hanba, M. Katsuhara, The photosynthetic response of tobacco plants overexpressing ice plant aquaporin McMIPB to a soil water deficit and high vapor pressure deficit. *J. Plant Res.* **126**, 517–527 (2013).

40. E. Ögren, J. R. Evans, Photosynthetic light-response curves. *Planta* **189**, 182–190 (1993).

41. C. A. Schneider, W. S. Rasband, K. W. Eliceiri, NIH Image to ImageJ: 25 years of image analysis. *Nat. Methods* **9**, 671–675 (2012).

42. K. Nishida, N. Kodama, S. Yonemura, Y. T. Hanba, Rapid response of leaf photosynthesis in two fern species *Pteridium aquilinum* and *Thelypteris dentata* to changes in CO_2 measured by tunable diode laser absorption spectroscopy. *J. Plant Res.* **128**, 777–789 (2015).

43. J. F. Thain, Curvature correction factors in the measurement of cell surface areas in plant tissues. *J. Exp. Bot.* **34**, 87–94 (1983).

44. C. Fu, W. P. Donovan, O. Shikapwashya-Hasser, X. Ye, R. H. Cole, Hot fusion: An efficient method to clone multiple DNA fragments as well as inverted repeats without ligase. *PLOS ONE* **9**, e115318 (2014).

45. Y. Benjamini, Y. Hochberg, Controlling the false discovery rate: A practical and powerful approach to multiple testing. *J. R. Stat. Soc. Ser. B Stat. Method.* **57**, 289–300 (1995).

46. K. Tamura, G. Stecher, S. Kumar, MEGA11: Molecular evolutionary genetics analysis version 11. *Mol. Biol. Evol.* **38**, 3022–3027 (2021).

47. J.-W. Choi, H. H. Choi, Y.-S. Park, M.-J. Jang, S. Kim, Comparative and expression analyses of AP2/ERF genes reveal copy number expansion and potential functions of ERF genes in Solanaceae. *BMC Plant Biol.* **23**, 48 (2023).

48. D. T. Jones, W. R. Taylor, J. M. Thornton, The rapid generation of mutation data matrices from protein sequences. *Bioinformatics* **8**, 275–282 (1992).

49. M. Meyer, U. Seibt, H. Griffiths, To concentrate or ventilate? Carbon acquisition, isotope discrimination and physiological ecology of early land plant life forms. *Philos. Trans. R. Soc. Lond. B Biol. Sci.* **363**, 2767–2778 (2008).

50. A. V. Perera-Castro, M. J. Waterman, S. A. Robinson, J. Flexas, Limitations to photosynthesis in bryophytes: Certainties and uncertainties regarding methodology. *J. Exp. Bot.* **73**, 4592–4604 (2022).

51. J. R. Evans, T. D. Sharkey, J. A. Berry, G. D. Farquhar, Carbon isotope discrimination measured concurrently with gas exchange to investigate CO_2 diffusion in leaves of higher plants. *Aust. J. Plant Physiol.* **13**, 281–292 (1986).

52. J. R. Evans, S. Von Caemmerer, Temperature response of carbon isotope discrimination and mesophyll conductance in tobacco. *Plant Cell Environ.* **36**, 745–756 (2013).

53. C. A. Roeske, M. H. O'Leary, Carbon isotope effects on the enzyme-catalyzed carboxylation of ribulose bisphosphate. *Biochemistry* **23**, 6275–6284 (1984).

54. Y. Tazoe, S. Von Caemmerer, M. R. Badger, J. R. Evans, Light and CO_2 do not affect the mesophyll conductance to CO_2 diffusion in wheat leaves. *J. Exp. Bot.* **60**, 2291–2301 (2009).

55. A. Laisk, O. Kiirats, V. Oja, Assimilatory power (postillumination CO_2 uptake) in leaves: Measurement, environmental dependencies, and kinetic properties. *Plant Physiol.* **76**, 723–729 (1984).

56. K. Takemura, R. Watanabe, R. Kameishi, N. Sakaguchi, H. Kamachi, A. Kume, I. Karahara, Y. T. Hanba, T. Fujita, Hypergravity of 10g changes plant growth, anatomy, chloroplast size, and photosynthesis in the moss *Physcomitrella patens*. *Microgravity Sci. Technol.* **29**, 467–473 (2017).

57. D. T. Hanson, K. Renzaglia, J. C. Villarreal, "Diffusion Limitation and CO_2 Concentrating Mechanisms in Bryophytes," in *Photosynthesis in Bryophytes and Early Land Plants*, T. D. Hanson, K. S. Rice, Eds. (Springer, 2014); http://dx.doi.org/10.1007/978-94-007-6988-5_6, pp. 95–111.
